# Cathepsin K Aggravates Pulmonary Fibrosis Through Promoting Fibroblast Glutamine Metabolism and Collagen Synthesis

**DOI:** 10.1002/advs.202413017

**Published:** 2025-07-03

**Authors:** Mengting Chen, Xiaoxiao Meng, Yong Zhu, Dapeng Wang, Mengmeng Wang, Ziyuan Wang, Xue Tian, Jiaxiang Zhang, Zhiying Yue, Zhengfeng Yang, Ruilan Wang

**Affiliations:** ^1^ Department of Critical Care Medicine Shanghai General Hospital Shanghai Jiaotong University School of Medicine Shanghai 201620 China; ^2^ Department of Intensive Medicine The Affiliated Wuxi People's Hospital of Nanjing Medical University Wuxi People's Hospital Wuxi Medical Center Nanjing Medical University Wuxi Jiangsu 214023 China; ^3^ Precision Research Center for Refractory Diseases Shanghai Jiao Tong University Pioneer Research Institute for Molecular and Cell Therapies Shanghai General Hospital Shanghai Jiao Tong University School of Medicine Shanghai 201620 China; ^4^ State Key Laboratory of Innovative Immunotherapy School of Pharmaceutical Sciences Shanghai Jiao Tong University Shanghai 200240 China

**Keywords:** collagen synthesis, CTSK, glutamine metabolism, pulmonary fibrosis, SNX9

## Abstract

Fibroblast collagen synthesis is a hallmark of the pathogenesis and progression of pulmonary fibrosis (PF). However, the factors that trigger the abnormal activation of fibroblasts in PF are still not well understood. Using proteomics and single‐cell sequencing dataset screening, extra accumulation of Cathepsin K (CTSK) is detected in the periphery as well as in fibroblasts in the lungs of PF mouse models. Addition of recombinant CTSK (rCTSK) aggravates collagen accumulation and PF progression in bleomycin‐induced PF mice. Mechanically, CTSK underwent endocytosis through interaction with sorting nexin 9 (SNX9), which is engaged in TGF‐β1 induced SMAD3 activation for downstream glutaminase 1 (GLS1) upregulation and glutamine enrichment. In turn, extra glutamine increases collagen synthesis in fibroblasts. More significantly, serum CTSK levels positively correlated with glutamine levels and poor prognosis in patients with PF. Thus, the results identify CTSK as a novel regulator of fibroblast activation that remodels glutamine metabolism and promotes collagen synthesis during PF pathogenesis. The correlation between peripheral CTSK and glutamine levels implies its future feasibility in the prediction and prevention of PF progression.

## Graphical abstract

CTSK plays a critical role in pulmonary fibrosis. Excessive CTSK accumulation interacts with SNX9 to enhance TGF‐β1‐induced SMAD3 activation and GLS1 expression in fibroblasts, driving glutamine metabolism for collagen biosynthesis and exacerbating pulmonary fibrosis. Mechanistically, CTSK activates a vicious cycle of collagen production in pulmonary fibrosis, highlighting its regulatory role and potential as a therapeutic target.

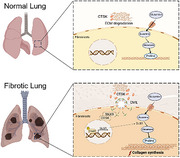

## 1 Introduction

Pulmonary fibrosis (PF) is a progressive and often fatal outcome of various lung diseases, with idiopathic pulmonary fibrosis (IPF) being a prominent subtype primarily driven by chronic lung inflammation.^[^ ^1^, ^2^ ^]^ The prognosis of patients with IPF remains poor, with an average survival of 3‒5 years post‐diagnosis.^[^ ^3^ ^]^ The incidence of PF is increasing worldwide, particularly in the older population.^[^ ^4^ ^]^ The COVID‐19 pandemic has dramatically increased the incidence of PF, reaching nearly 2‒6%.^[^ ^5^ ^]^ ≈44.9% of the COVID‐19 survivors develop PF,^[^ ^6^ ^]^ marking a major shift in the disease landscape. However, at present, clinical treatments for PF are of low efficacy, with only pirfenidone and nintedanib.^[^ ^7^ ^]^ Consequently, elucidating the mechanisms underlying PF is crucial for the development of novel therapeutic strategies.^[^ ^8^ ^]^

Fibroblasts provide a structural basis for the lungs and remodel the local environment of lung tissues because of their ability to proliferate, undergo fibroblast‐to‐myofibroblast transition (FMT), and exacerbate extracellular matrix (ECM) accumulation.^[^ ^9^ ^]^ Repeated and severe lung injury promotes fibroblast proliferation through functional modulation.^[^ ^10^ ^]^ Lung‐resident macrophages, epithelial cells, and endothelial cells establish an injury‐related microenvironment with abnormal cytokines, growth factors, and chemokines, which drives fibroblast overactivation.^[^ ^11^ ^]^ This ultimately leads to irreversible destruction of the lung architecture and the development of PF.^[^ ^12^ ^]^ Therefore, understanding the molecular mechanisms governing fibroblast behavior in PF may provide effective therapeutic targets for this devastating disease.

Matrix metalloproteinases (MMPs) are well‐characterized enzymes involved in ECM degradation,^[^ ^13^, ^14^ ^]^ and play both beneficial and detrimental roles in disease progression.^[^ ^15^ ^]^ Similarly, cathepsins (CTSs), another family of proteolytic enzymes, are implicated in ECM remodeling and have emerged as potential therapeutic targets for ECM‐related diseases, including osteoporosis, cancer, and cardiovascular diseases.^[^ ^16^ ^]^ Among the CTS family members, CTSS and CTSK are the most promising targets for certain inhibitors in advanced clinical trials.^[^ ^17^ ^]^ They also have a dual function in the modulation of fibrosis. CTSS is mainly secreted by the macrophages, and can promote liver fibrosis and PF.^[^ ^18^, ^19^ ^]^ However, the role of CTSK in the pathogenesis of PF remains unclear. CTSK is correlated with both collagen synthesis and degradation in pulmonary tuberculosis lesions^[^ ^20^ ^]^ while some studies suggest it may promote PF.^[^ ^21^, ^22^, ^23^ ^]^ Owing to the complexity of CTSs in mediating PF, their precise mechanisms warrant further investigation.

In the present study, we screened for excessive accumulation of CTSK in the periphery and fibroblasts of PF mouse models using proteomic and single‐cell RNA‐sequencing (scRNA‐seq) analyses. Addition of CTSK aggravated PF in bleomycin (BLM)‐induced fibrosis mice, as evidenced by increased collagen deposition in lung tissue. Mechanically, CTSK interacted with sorting nexin 9 (SNX9) undergoing endocytosis and was engaged in TGF‐β1 induced SMAD3 activation for downstream glutaminase upregulation and collagen biosynthesis in the fibroblasts. We also demonstrated that glutamine promotes collagen synthesis in fibroblasts. More significantly, serum CTSK levels positively correlated with glutamine content and poor prognosis in patients with PF. Thus, our study provides direct evidence that CTSK acts as a novel regulatory factor in fibroblast activation, identifying both CTSK and glutamine as potential therapeutic targets for PF.

## 2 Results

### 2.1 Proteomics Combined with scRNA‐Seq Analysis Identifies CTSK Enrichment in the Fibroblasts During PF Progression

**Figure 1:**
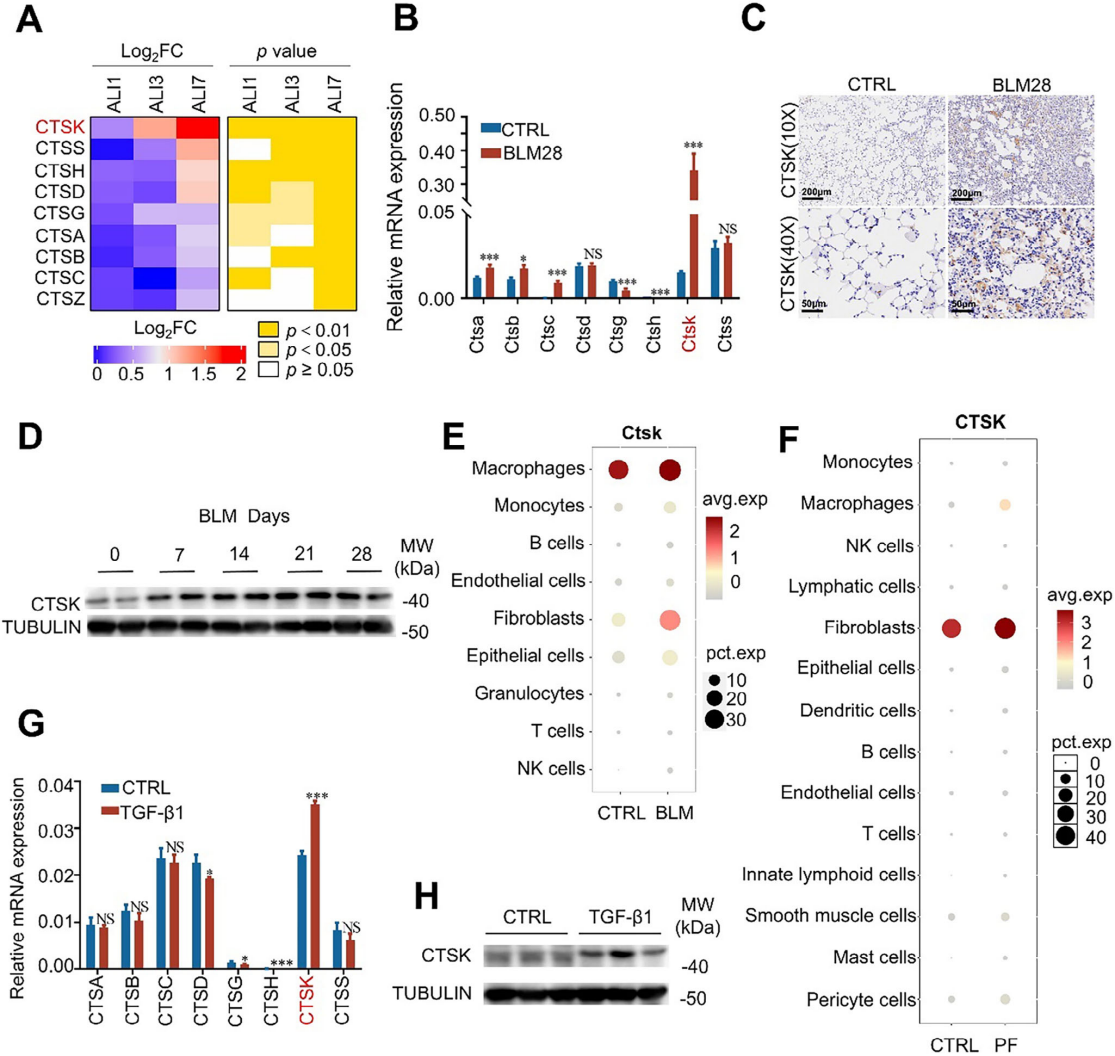
CTSK is accumulated along with the progression of PF. A) Proteomic analysis on the expression levels of CTS family members, including Ctsk, Ctss, Ctsh, Ctsd, Ctsg, Ctsa, Ctsb, Ctsc, Ctsz in the lung tissues from PQ‐induced ALI mice model. Briefly, mice were treated with PQ and the lung tissues were collected on day 1 (ALI 1), day 3 (ALI 3), and day 7 (ALI 7) respectively for proteomic analysis. B) Comparison of mRNA expression levels of *Ctsa*, *Ctsb*, *Ctsc, Ctsd*, *Ctsg*, *Ctsh, Ctsk*, and *Ctss* in the lung tissues between control (CTRL) and BLM‐treated mice. C) Immunohistochemical analysis on the abundance and sub‐localization of CTSK in the lung tissues from CTRL and BLM‐treated mice. D) Detection of protein abundance of CTSK in the lung tissues from BLM‐treated mice at Day 0, 7, 14, 21, and 28 respectively by Western blotting. E) Bubble plots indicating the presence of *Ctsk* in 9 cell types from the lung tissues of CTRL and BLM‐induced mice derived from the scRNA‐seq dataset GSE111664. F) Bubble plots indicating the presence of *Ctsk* in 14 cell types from lung tissues of healthy (CTRL) and PF patients derived from the scRNA‐seq dataset GSE136831. G) Detection of mRNA expression levels of *Ctsa*, *Ctsb*, *Ctsc*, *Ctsd*, *Ctsg*, *Ctsh*, *Ctsk*, and *Ctss* in CTRL and TGF‐β1 treated MRC‐5 cells. H) Determination of CTSK protein levels in CTRL and TGF‐β1 treated MRC‐5 cells by Western blotting. Data were presented as mean ± standard deviation (SD). *: *p* < 0.05, **:*p* < 0.01, ***: *p* < 0.001, NS, not statistically significant by the Student's *t*‐test. Unless indicated, data were representative of two independent experiments.

We established a paraquat (PQ)‐induced acute lung injury (ALI) mouse model and collected lung tissues at different time points for proteomic analysis. In particular, we found that the CTS family members, including CTSA, CTSB, CTSC, CTSD, CTSG, CTSH, CTSK, CTSS, and CTSZ, were gradually upregulated during the progression of PQ‐induced ALI, among which CTSK exhibited the most significant accumulation (**Figure** **1**A; Figure S1A–I, Supporting Information). Only two matrix metalloproteinase family members, TIMP3 and MMP3, were detectable with no sustained accumulation (Figure S1J,K, Supporting Information). We further detected the expression of CTS family members in the lung tissues of mice with BLM‐induced PF. Consistent with the results from PQ‐induced ALI, *CTSK* was dramatically upregulated in the lung tissues of BLM‐induced PF mice (Figure 1B) compared with those from control mice. This upregulation was validated at the protein level by immunohistochemical assays (Figure 1C) and western blotting (Figure 1D). CTSK expression gradually increased after BLM induction, with a peak on day 21, coinciding with the onset of severe PF in the model.^[^ ^24^ ^]^

To further define the role of CTSK in lung tissues, we used published scRNA‐seq data from mice with BLM‐induced PF (GSE111664) to identify the expression patterns of CTS family members in fibrotic lung tissues. *CTSK* was specifically upregulated in macrophages and fibroblasts (Figure 1E; Figure S2, Supporting Information), with a dramatic increase in the number of fibroblasts. Consistently, *CTSK* was also upregulated in fibroblasts from patients with PF, based on scRNA‐seq data from GSE136831 (Figure 1F).

We have treated fibroblast cell line MRC‐5 cells with TGF‐β1 to mimic FMT, which is an irreversible step of PF occurrence. We found that CTSK was dramatically upregulated at both mRNA and protein levels upon TGF‐β1 stimulation (Figure 1G,H). Collectively, our results indicate that CTSK is enriched in fibroblasts following severe lung injury and PF.

### 2.2 CTSK Accumulation Enhances Collagen Synthesis in the Fibroblasts

**Figure 2:**
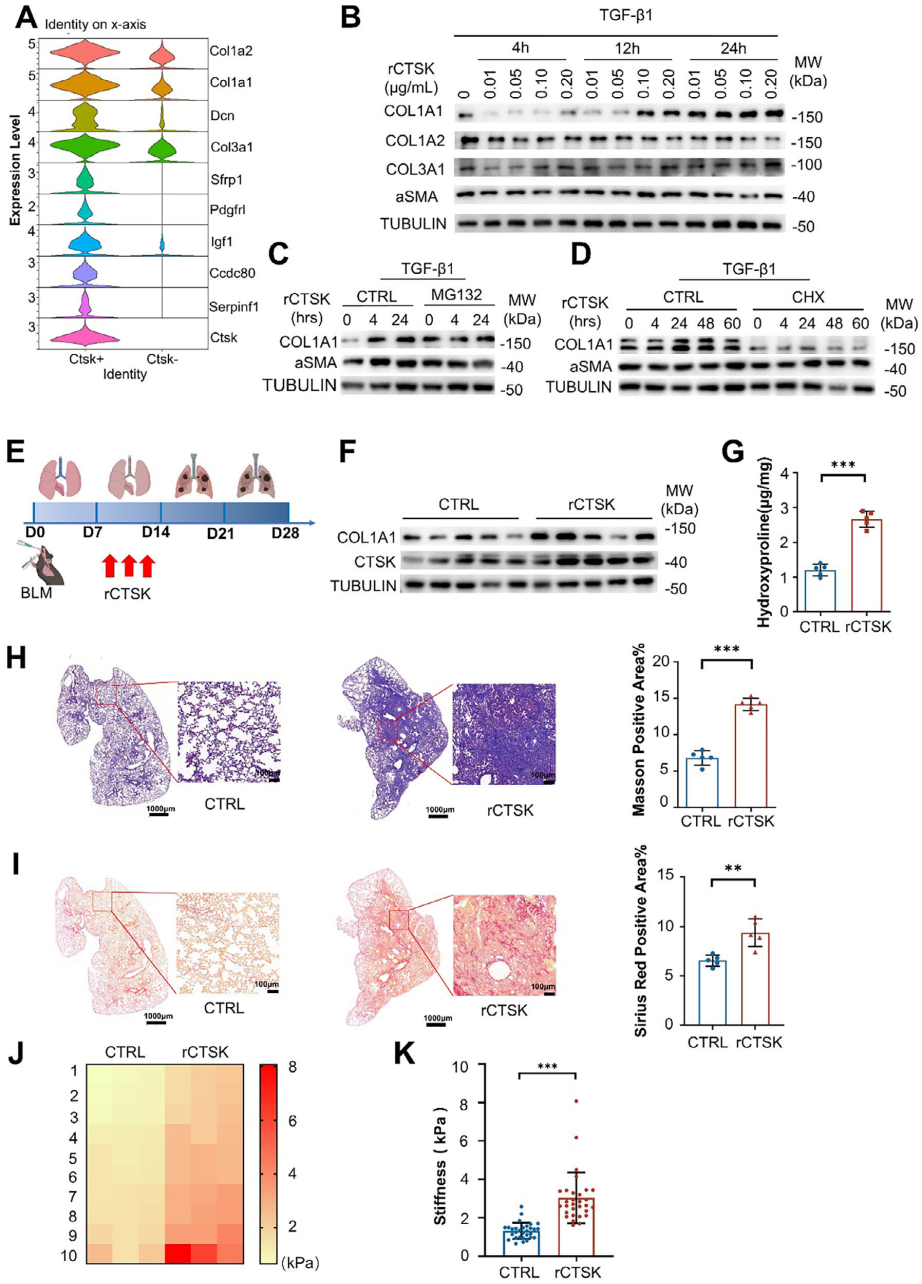
CTSK aggravates the severity of PF. A) Violin plots showed top10 marker genes in *Ctsk* ^+^ and *Ctsk* ^−^ groups in collagen‐producing cells based on the scRNA‐seq dataset GSE132771. B) Detection of expression levels of COL1A1, COL1A2, COL3A1 and αSMA in MRC‐5 cells treated with TGF‐β1 followed by the addition of 0.02, 0.05, 0.1, 0.2 µg mL^−1^ recombinant (rCTSK) for 4, 12 and 24 h respectively by Western blotting. C) Detection of expression levels of COL1A1 and αSMA in TGF‐β1‐treated MRC‐5 cells in the presence of rCTSK (0.1 µg mL^−1^) with or without MG132 (10 µm) for 4 and 24 h by Western blotting. D) Detection of expression levels of COL1A1 and αSMA in TGF‐β1‐treated MRC‐5 cells in the presence of rCTSK (0.1 µg mL^−1^) with or without cycloheximide (CHX) (10 µg/mL) for 0, 4, 24, 48 and 60 h by Western blotting. E) Experimental diagram of BLM‐induced PF mice models with the addition of rCTSK intratracheally at Day 7, 9, and 11 respectively. Mice were sacrificed at Day 28 and subjected to further experiments. (F) Expression levels of COL1A1 in the lung tissues between CTRL and rCTSK‐treated mice after BLM exposure by Western blotting. G) Comparisons of hydroxyproline contents in the lung tissues between CTRL and rCTSK treated mice after BLM exposure by ELISA (n = 5 per group). H) Masson's staining of the lung tissues in CTRL (left) and rCTSK (middle) treated‐mice after BLM exposure with statistical comparisons of the percentages of Masson positive areas (right) (n = 5 per group). Scale bar: 1 mm and 100 µm respectively. I) Sirius red staining of the lung tissues in CTRL (left) and rCTSK (middle) treated mice after BLM exposure with statistical comparison of the percentages of Sirius red positive areas (right) (n = 5 per group). Scale bar: 1 mm and 100 µm respectively. J) The typical mechanical  heatmaps of 30 detected positions of CTRL and rCTSK treated mice by AFM. K) Quantification of tissue stiffness in CTRL and rCTSK‐treated mice based on AFM measurements. Data were presented as mean ± standard deviation (SD). *: *p* < 0.05, **:*p* < 0.01, ***: *p* < 0.001, NS, not statistically significant by the Student's *t*‐test. Unless indicated, data were representative of two independent experiments.

To understand the potential functions of upregulated CTSK in PF progression, we performed further analysis of PF scRNA‐seq data from normal and fibrotic lungs in mice (GSE132771)^[^ ^25^ ^]^ by categorizing collagen‐producing cells. The analysis involved categorizing collagen‐producing cells, which are key contributors to PF, into *Ctsk*‐positive and *Ctsk*‐negative subsets. Our results revealed that collagen component genes, including *Col1a1*, *Col1a2*, and *Col3a1*, were strikingly enriched in *Ctsk* positive fibroblasts compared to their *Ctsk* negative counterparts (**Figure** **2**A), indicating that CTSK might promote collagen synthesis and PF progression.

Thus, we hypothesized that the upregulation of CTSK following lung injury may serve as a rapid response to induce fibrotic stress. To address this possibility, we stimulated the fibroblast cell line MRC‐5 with TGF‐β1 and treated the cells with rCTSK at varying concentrations and durations, and monitored the abundance of collagen component genes. We found that rCTSK specifically upregulated the expression of COL1A1 at high concentrations (0.1 µg/mL) and with prolonged stimulation (24 h), while the expression of COL1A2, COL3A1, and α‐SMA remained unaffected (Figure 2B). These results were corroborated in primary mouse lung fibroblasts (Figure S3A, Supporting Information), suggesting that excessive rCTSK stimulation specifically enhanced COL1A1 protein expression.

To clarify how rCTSK modulates collagen synthesis at the protein level, we treated MRC‐5 cells with the proteasomal inhibitor MG132 during induction with TGF‐β1 and rCTSK. Although MG132 increased COL1A1 levels under basal conditions, it did not promote rCTSK‐triggered COL1A1 production (Figure 2C). Conversely, treatment with the protein synthesis inhibitor, cycloheximide significantly suppressed both basal and rCTSK‐induced COL1A1 production (Figure 2D). Notably, α‐SMA levels remained unchanged regardless of the inhibitor treatment. These results indicated that rCTSK promotes COL1A1 synthesis rather than inhibiting its degradation.

Consistent with the in vitro findings, we administered rCTSK to mice with BLM‐induced PF and monitored fibrosis progression on day 28 (Figure 2E). We observed increased COL1A1 production and hydroxyproline levels in the lung tissues of BLM‐induced mice treated with rCTSK (Figure 2F,G). Masson's trichrome and Sirius Red staining revealed enhanced collagen deposition and additional collagen fibers in rCTSK‐treated lungs (Figure 2H,I). Furthermore, mRNA levels of fibrosis markers, including *Col1a1*, *αsma*, *Eln*, and *Fn*, were significantly elevated in rCTSK‐treated lungs (Figure S3B–E, Supporting Information). Atomic force microscopy (AFM) further confirmed increased lung stiffness in rCTSK‐treated mice compared to controls (Figure 2J,K).

To further investigate the temporal effects of excessive rCTSK stimulation on fibrosis progression, we collected lung tissue and serum on days 0, 7, 14, and 21 after BLM induction. We observed that rCTSK administration on day 7 significantly elevated Ctsk and Col1a1 protein expression in lung tissues harvested on days 14 and 21 (Figure S3F, Supporting Information). In addition, serum CTSK levels were markedly higher in rCTSK‐treated mice at these time points (Figure S3G, Supporting Information). Collectively, these findings suggest that excessive CTSK expression following lung injury exacerbates PF.

To further understand the importance of CTSK in PF progression, we analyzed PF progression in patients with CTSK deficiency using two approaches. We treated mice with odanacatib (ODN),^[^ ^26^ ^]^ a CTSK inhibitor, and examined the progression of PF (Figure S4A, Supporting Information). All indicators, including the abundance of collagen 1a1 protein (Figure S4B, Supporting Information) and hydroxyproline levels (Figure S4C, Supporting Information), morphological changes, (Figure S4D,E, Supporting Information), and extracellular matrix accumulation (Figure S4D,E, Supporting Information) were significantly increased in the ODN‐treated lung tissues. These observations suggest that restriction of CTSK activity aggravates BLM‐induced PF progression.

Next, we generated fibroblast‐specific CTSK conditional knockout mice (Col1a2‐CreERT; CTSK^flox/flox^) (Figure S4F, Supporting Information). The abundance of the collagen 1a1 protein (Figure S4G, Supporting Information) was upregulated in the CTSK‐deficient group. The higher levels of hydroxyproline (Figure S4H, Supporting Information), more severe damage to lung architecture, including disappearance of alveolar structure, thickening of pulmonary septa, accumulation of extracellular matrix, and presence of numerous collagen fiber deposits, was observed in CTSK‐deficient mice compared to control mice (Figure S4I,J, Supporting Information). These results together suggest that CTSK deficiency in fibroblast aggravates BLM‐induced PF progression, which is consistent with the reported enzymatic function of CTSK in degradation of extracellular matrix in multiple tissues.^[^ ^27^ ^]^

Nevertheless, our data indicate a dual role of CTSK in PF, contingent on its source and concentration. While endogenous CTSK exerted anti‐fibrotic effects consistent with its canonical role in collagen degradation, high doses of exogenous rCTSK unexpectedly promoted fibrosis.

### 2.3 CTSK Interacts with SNX9 to Promote Collagen Synthesis

**Figure 3:**
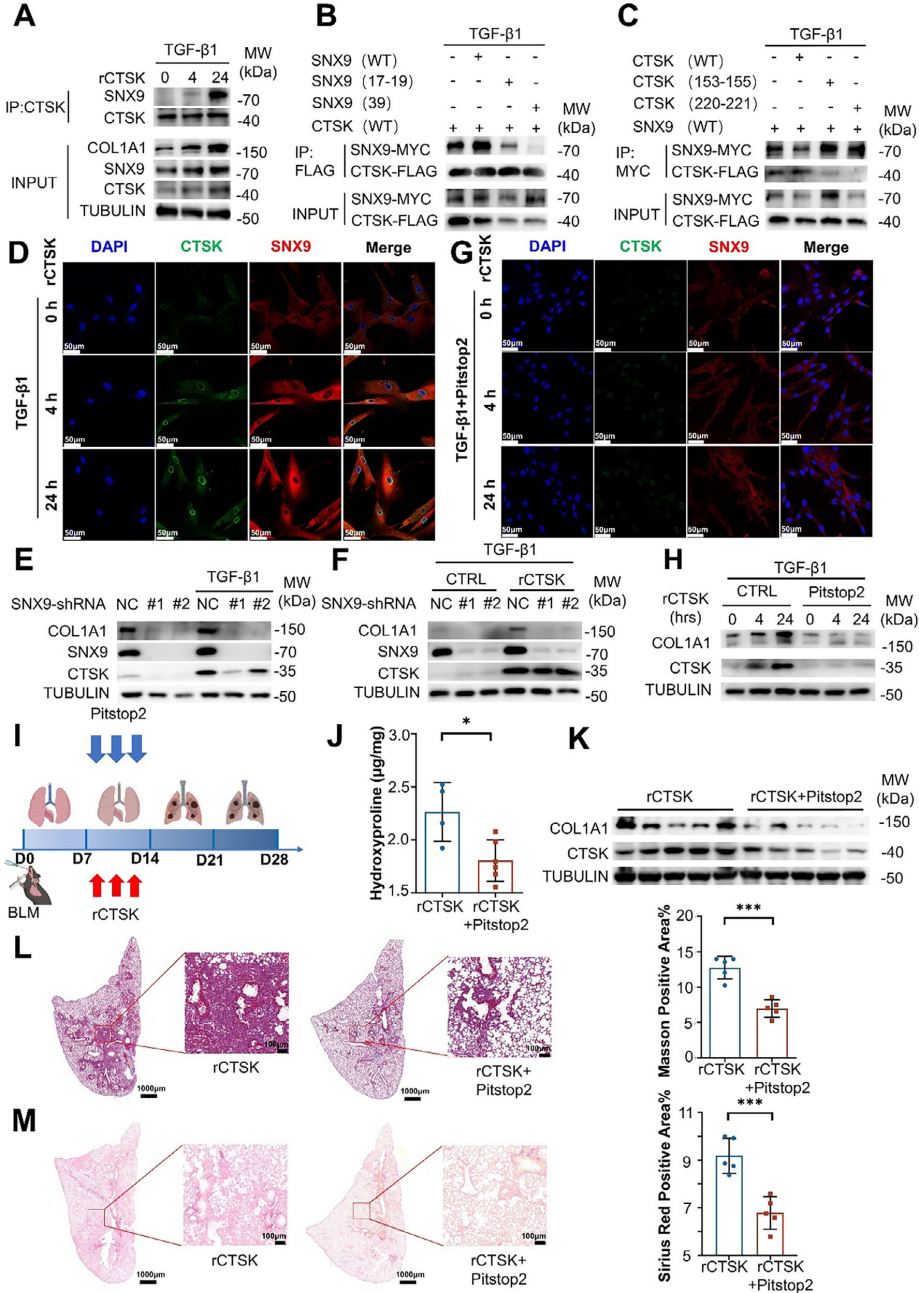
SNX9 mediates CTSK endocytosis to promote collagen synthesis. A) Co‐IP validation for CTSK‐SNX9 interaction. Briefly, MRC‐5 cells were treated with TGF‐β1 and rCTSK (0.1 µg mL^−1^) for 0, 4, or 24 h. IP was conducted with anti‐His antibody (5 µg mg^−1^ lysate). Detecting Abs: anti‐SNX9 and anti‐CTSK Abs. Lysate input: COL1A1, SNX9, CTSK, and TUBULIN. B,C) Co‐IP validation for CTSK‐SNX9 predicted interaction in the condition with 10 ng mL^−1^ of TGF‐β1 stimulation for 24 h in MRC‐5 cells. D) Detection of colocalization of CTSK and SNX9 in MRC‐5 cells treated with TGF‐β1 and rCTSK (0.1 µg mL^−1^) for 0, 4, or 24 h by immunofluorescence staining. Scale bars: 50 µm. E) Detection of expression levels of COL1A1, SNX9, and CTSK in MRC‐5 cells in the presence of scrambled or *SNX9* shRNA upon TGF‐β1 stimulation. F) Detection of expression levels of COL1A1, SNX9, and CTSK in MRC‐5 cells in the presence of scrambled or *SNX9* shRNA infected upon TGF‐β1 stimulation with or without rCTSK (0.1 µg mL^−1^). G) Colocalization of CTSK and SNX9 in MRC‐5 cells upon TGF‐β1 and rCTSK (0.1 µg mL^−1^) stimulation for 0, 4, or 24 h with an inhibitor of SNX9 Pitstop2 by immunofluorescence staining. Scale bars: 50 µm. H) Detection of expression levels of COL1A1, SNX9, and CTSK in MRC‐5 cells upon TGF‐β1 and rCTSK (0.1 µg mL^−1^) stimulation for 0, 4, or 24 h with or without Pitstop2 (30 µm) by Western blotting. I) Experiment diagram of BLM‐induced PF mice models with the addition of rCTSK intratracheally at Day 7, 9, and 11 respectively. Pitstop2 was intratracheally injected on Days 8, 10, and 12 respectively. Mice were sacrificed at Day 28 and subjected to further experiments. J) Detection of hydroxyproline contents in the lung tissues from rCTSK or rCTSK + Pitstop2 treated mice after BLM exposure by ELISA. (n = 5 per group). K) Detection of the abundance of COL1A1 and CTSK in the lung tissues from rCTSK or rCTSK + Pitstop2 treated mice after BLM exposure by Western blotting. K) Masson's staining of the lung tissues from rCTSK or rCTSK + Pitstop2 treated mice after BLM exposure with statistical comparison. Scale bar, 1 mm (left) and 100 µm (right). (n = 5 per group). L) Sirius red staining of lung tissues from rCTSK or rCTSK + Pitstop2 treated mice after BLM exposure with statistical comparison. Scale bar, 1 mm (left) and 100 µm (right). (n = 5 per group). Data were presented as mean ± standard deviation (SD). *: *p* < 0.05, **:*p* < 0.01, ***: *p* < 0.001, NS, not statistically significant by the Student's *t*‐test. Unless indicated, data were representative of two independent experiments.

As mentioned above, previous studies have reported that CTSK is a secreted protease that degrades the extracellular matrix.^[^ ^20^ ^]^ However, our results revealed that pathologically accumulated CTSK promotes collagen synthesis in fibroblasts, implying that CTSK exerts an alternative modulation pattern, beyond its enzymatic activity during PF. To explore the relevant underlying mechanisms, we first identified proteins that might interact with CTSK. The proteomic profiles of MRC‐5 cells treated with TGF‐β1 and rCTSK for 0, 4, or 24 h were determined using LC‐MS/MS assays. Based on the criteria we have established, we identified SNX9 as one of the most potential candidates (Figure S5A, Supporting Information). SNX9 has been reported to be involved in clathrin‐mediated endocytosis (CME) as well as intracellular trafficking.^[^ ^28^ ^]^ We further verified direct association between CTSK and SNX9 by Co‐IP in MRC‐5 cells upon in vitro treatment with TGF‐β1 and rCTSK. SNX9 directly interacted with CTSK, while COL1A1 and SNX9 apparently accumulated with the addition of CTSK in MRC‐5 cells (**Figure** **3**A).

We further analyzed the critical amino acid residues involved in the interaction between CTSK and SNX9. Previous studies have demonstrated that SNX9 is a multi‐domain protein, and its SH3 domain serves as a protein‐protein interaction module.^[^ ^28^ ^]^ We used AlphaFold3 to predict the potential binding interface between the SH3 domains of SNX9 and CTSK. Further analysis indicated that the amino acid residues at positions 155, 220, and 221 of CTSK (LYS155, LYS220, and CYS221, respectively) and amino acid residues at positions 17, 19, and 39 of SNX9 (ASN17, GLU19, TRP39) may constitute critical interaction sites (Figure S5B, Supporting Information). This prediction was subsequently validated through alanine screening mutagenesis together with Co‐IP analysis, with LYS220/CYS221 of CTSK and TRP39 of SNX9 being the most critical residues for the interaction, confirming the physical association between CTSK and SNX9 (Figure 3B,C). The immunofluorescence assay further revealed that CTSK colocalized with SNX9 mainly in the cytosol and on the nuclear membrane (Figure 3D), implying that rCTSK could be internalized by interacting with SNX9, followed by endocytosis. When we knocked down *SNX9* expression in the MRC‐5 cells, we found that COL1A1 expression was reduced in MRC‐5 cells upon TGF‐β1 induction (Figure 3E). Moreover, addition of rCTSK in *SNX9*‐knockdown MRC‐5 cells failed to increase COL1A1 production upon TGF‐β1 induction (Figure 3F).

To further investigate the roles of SNX9‐mediated endocytosis in promoting collagen synthesis by CTSK, we treated MRC‐5 cells with Pitstop2 (an inhibitor of CME^[^ ^29^ ^]^) upon TGF‐β1 induction. We observed reduced accumulation of CTSK in MRC‐5 cells (Figure 3G). Consistently, western blotting showed that COL1A1 levels in TGF‐β1‐induced MRC‐5 cells were also reduced when treated with Pitstop2 and rCTSK (Figure 3H). Furthermore, we treated BLM‐treated mice with rCTSK or rCTSK and Pitstop2 (Figure 3I) to investigate the in vivo effects of SNX9 endocytosis on PF progression. We found that hydroxyproline levels in the lung tissues of rCTSK plus Pitstop2 treated mice were dramatically decreased compared to those in rCTSK‐treated mice (Figure 3J). Both COL1A1 and CTSK protein levels in lung tissues were also downregulated in Pitstop2 treated BLM‐induced PF mice with the addition of rCTSK compared to those without Pitstop2 (Figure 3K). Both collagen and fiber deposition were alleviated when Pitstop2 was added according to Masson's and Sirius Red staining, respectively (Figure 3L‐M). Similarly, we observed a decrease in mRNA expression of fibrosis markers including *Col1a1, αsma, Eln*, and *Fn* when Pitstop2 was added (Figure S5C–F, Supporting Information). These results strongly suggest that CTSK interacts with SNX9 to undergo endocytosis, which promotes COL1A1 production in fibroblasts during PF.

### 2.4 rCTSK Facilitates SNX9‐Mediated SMAD3 Activation

**Figure 4:**
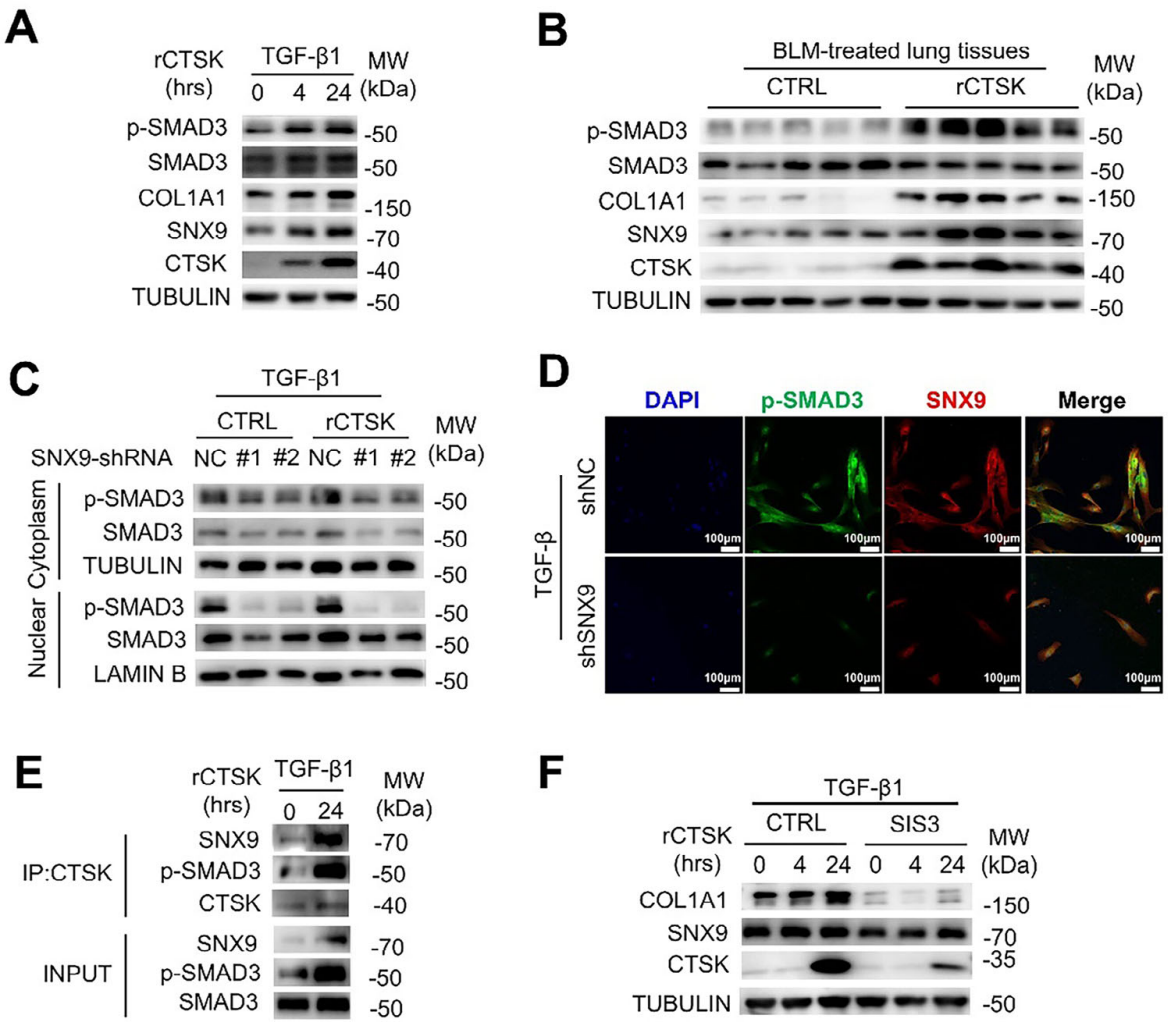
CTSK‐triggered collagen synthesis depends on TGF‐β1‐SMAD3 signaling. A) Detection of the abundance of p‐SMAD3, SMAD3, COL1A1, SNX9, and CTSK in MRC‐5 cells upon TGF‐β1 stimulation with rCTSK (0.1 µg mL^−1^) for 0, 4, or 24 h respectively by Western blotting. B) Detection of the abundance of p‐SMAD3, SMAD3, COL1A1, SNX9, and CTSK in the lung tissues from BLM‐induced mice with or without rCTSK treatment by Western blotting. C) Detection of the abundance of p‐SMAD3 and SMAD3 in the cytoplasmic or nuclear fractions of MRC‐5 cells in the presence of scrambled or *SNX9* shRNA infected upon TGF‐β1 stimulation with or without rCTSK by Western blotting. TUBULIN and LAMIN B were internal controls to represent the purity of cytoplasmic and nuclear fractions, respectively. D) Immunofluorescence analysis to determine the subcellular distribution of SNX9 and p‐SMAD3 in MRC‐5 cells upon TGF‐β1 stimulation with scrambled shRNA (shNC) or *SNX9* shRNA (shSNX9) transfection. Scale bars: 100 µm. E) Co‐IP validation for CTSK, SNX9, and p‐SMAD3 interaction. MRC‐5 cells were treated with or without TGF‐β1 and rCTSK (0.1 µg mL^−1^) for 24 h. F) Detection of the abundance of COL1A1, SNX9, and CTSK in MRC‐5 cells upon TGF‐β1 stimulation with (0.1 µg mL^−1^) or rCTSK plus SIS3 (10 µm) for 0, 4, or 24 h by Western blotting.

Next, we investigated the signaling pathways involved in CTSK‐SNX9 initiated COL1A1 production during PF progression. Previous studies have highlighted the role of TGF‐β1‐SMAD3 activation in FMT and subsequent PF progression.^[^ ^30^ ^]^ SNX9 has been demonstrated to promote SMAD3 phosphorylation and nuclear translocation.^[^ ^31^ ^]^ Therefore, we hypothesized that endocytic rCTSK facilitated SMAD3 activation through SNX9 for COL1A1 production. When we induced MRC‐5 cells with TGF‐β1, addition of rCTSK significantly increased SMAD3 phosphorylation accompanied with COL1A1 production (**Figure** **4**A). We further analyzed SMAD3 phosphorylation in the lung tissues of BLM‐treated mice treated with or without rCTSK. SMAD3 phosphorylation was upregulated upon COL1A1 and SNX9 overexpression (Figure 4B). Knockdown of *SNX9* attenuated rCTSK‐enhanced phospho‐SMAD3 in the cytosol and led to dramatic reduction of phosphor‐SMAD3 accumulation in the nucleus of MRC‐5 cells treated by TGF‐β1 based on western blotting (Figure 4C) and immunofluorescence assays (Figure 4D). Co‐IP revealed an association between CTSK, SNX9, and phosphorylated SMAD3 (Figure 4E). These results suggest that the CTSK‐SNX9 interaction enhances the activation of phosphorylated SMAD3. Next, we used the SMAD3 inhibitor SIS3 to evaluate SMAD3 activation during COL1A1 production. We found that SIS3 significantly inhibited basal and rCTSK‐enhanced COL1A1 production (Figure 4F). Interestingly, the intracellular CTSK levels were also reduced. These results thus indicate that rCTSK facilitates TGF‐β1‐induced SMAD3 activation via SNX9.

### 2.5 CTSK Facilitates GLS1 Expression and Glutamine Metabolism Relying on SNX9‐Mediated SMAD3 Activation

**Figure 5:**
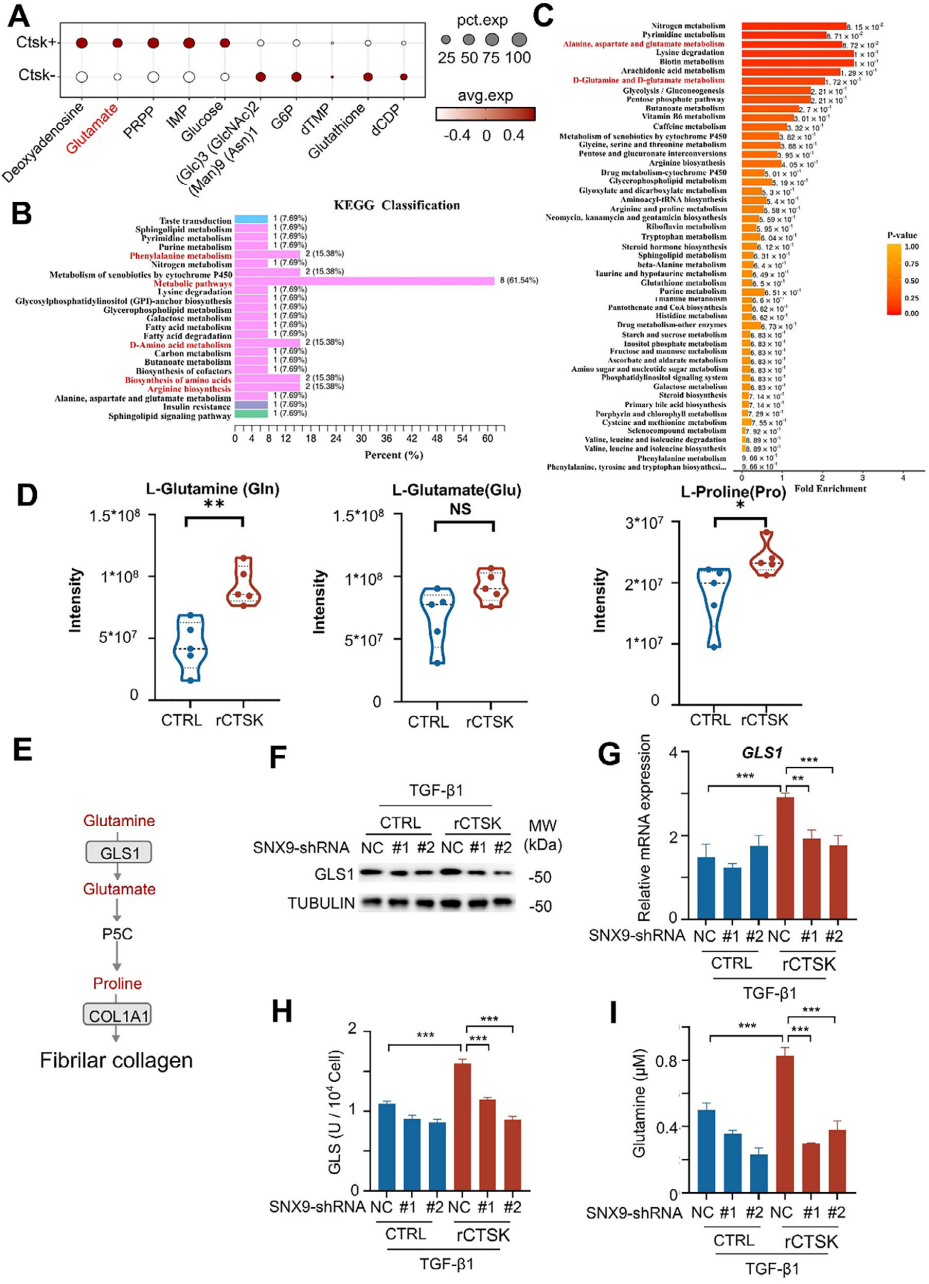
Excessive CTSK exaggerates glutamine metabolism. A) Bubble plots showing top10 metabolites in *CTSK* ^+^ and *CTSK* ^−^ groups in PF patients based on the scRNA‐seq dataset GSE136831 using scFEA. B,C) MRC‐5 cells were stimulated with TGF‐β1 with or without rCTSK (0.1 µg mL^−1^) for 24 h and subjected to transcriptome and metabolomic assays. Kyoto Encyclopedia of Genes and Genomes (KEGG) pathway enrichment analysis was performed based on transcriptome B) and Metabolite Set Enrichment Analysis (MSEA) was performed to define metabolite profiles difference between TGF‐β1 alone and TGF‐β1 plus rCTSK treated MRC‐5 cells. (n = 4). D) Detection of L‐Glutamine, L‐Glutamate, and L‐Proline levels in MRC‐5 cells upon TGF‐β1 stimulation with or without rCTSK (0.1 µg mL^−1^) for 24 h by targeted metabolic assay. E) Scheme of glutamine transport and catabolic pathways. F,G) Determination of the protein F) and mRNA expression level G) of GLS1 in scrambled shRNA (NC) or *SNX9* shRNA transfected MRC‐5 cells upon TGF‐β1 stimulation with or without rCTSK (0.1 µg mL^−1^) for 24 h. H,I) Determination of GLS1 activity H) and the glutamine concentration I) in scrambled shRNA (NC) or *SNX9* shRNA transfected MRC‐5 cells upon TGF‐β1 stimulation with or without rCTSK (0.1 µg mL^−1^). Data were presented as mean ± standard deviation (SD). *:*p* < 0.05, **:*p* < 0.01, ***:*p* < 0.001, NS, not statistically significant by the Student's *t*‐test. Unless indicated, data were representative of two independent experiments.

Collagen synthesis requires amino acid blocks derived from metabolic pathways.^[^ ^32^ ^]^ When we analyzed scRNA‐seq data from patients with PF (GSE136831) using scFEA and divided the fibroblasts into *CTSK*‐positive and *CTSK*‐negative groups, we found that glutamate was significantly enriched in *CTSK*‐positive fibroblasts (**Figure** **5**A). We further performed untargeted metabolomics assays on TGF‐β1 induced MRC‐5 cells with or without rCTSK treatment to define the metabolic profiles affected by CTSK through LC‐MS/MS. As expected, KEGG analysis showed that rCTSK stimulation of TGF‐β1–induced MRC‐5 cells significantly enriched metabolic pathways, with four amino acid‐related pathways (biosynthesis of amino acids, arginine biosynthesis, D‐amino acid metabolism, and phenylalanine metabolism) accounting for 15.38% (Figure 5B). MSEA enrichment analysis further showed that the glutamine metabolic pathway, which is a key pathway for proline and glycine generation for collagen biosynthesis, was among the top activated metabolic pathways and was significantly enriched upon rCTSK stimulation (Figure 5C). To further verify the effect of CTSK on the activation of glutamine metabolic pathway, we performed targeted metabolomics assays on TGF‐β1 induced MRC‐5 cells with or without rCTSK treatment. The results revealed that seven amino acid levels were elevated rCTSK‐treated cells than control cells where the increase in glutamine and proline were more dramatic (Figure 5D; Figure S6, Supporting Information).

Glutaminase 1 (GLS1) is a key enzyme involved in the conversion of glutamine into glutamate. Glutamate is metabolized to 1‐pyrroline‐5‐carboxylate (P5C) by ALDH18A1. P5C is catalytically converted to proline by P5C reductase (PYCR) to produce COL1A1 and fibrillar collagen (Figure 5E).^[^ ^33^ ^]^ TGF‐β1 signal is demonstrated to promote GLS1 expression and enhance glutamine metabolism.^[^ ^34^ ^]^ While rCTSK treatment increased GLS1 expression in MRC‐5 cells upon TGF‐β1 stimulation, interference with *SNX9* expression largely reduced the rCTSK‐enhanced GLS1 expression at the protein (Figure 5F) and mRNA levels (Figure 5G). Consistently, rCTSK also enhanced GLS1 enzyme activity in TGF‐β1‐stimulated MRC‐5 cells, whereas *SNX9* knockdown led to a reduction in GLS1 enzyme activity, similar to the rCTSK‐unstimulated levels (Figure 5H), supporting the role of CTSK in promoting GLS1 expression and activity. Furthermore, we detected an abundance of glutamine in TGF‐β1‐stimulated MRC‐5 cells after rCTSK treatment. However, suppression of *SNX9* expression by shRNA in TGF‐β1‐stimulated MRC‐5 cells reduced glutamine content (Figure 5I). These findings cumulatively substantiate our hypothesis that rCTSK facilitates activation of the glutamine metabolic pathway by enhancing GLS1 expression, which ultimately leads to excessive COL1A1 production.

### 2.6 CTSK‐Triggered Glutamine Catabolism Mediates Collagen Overproduction in the Fibroblasts

**Figure 6:**
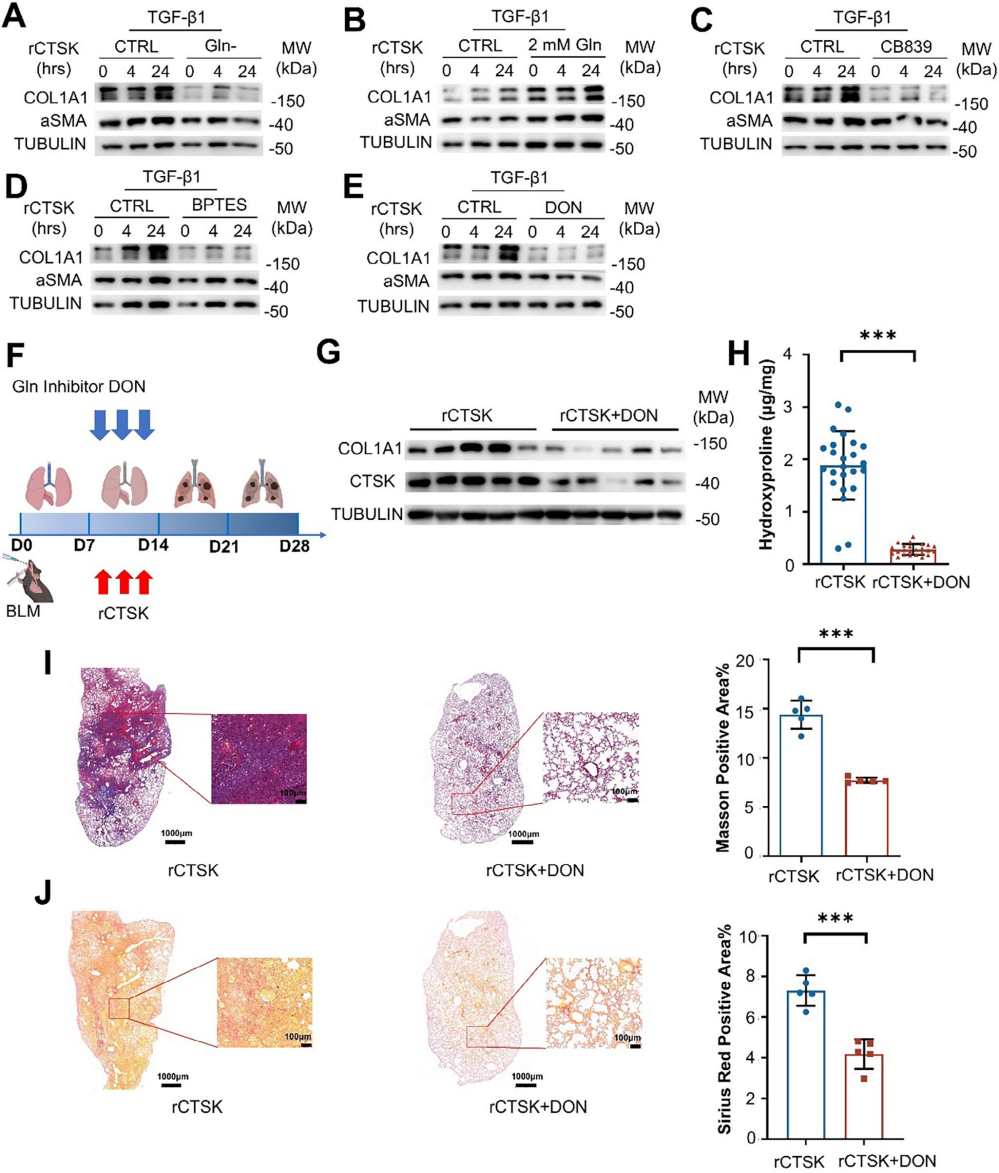
CTSK‐triggered glutamine metabolism promotes collagen synthesis in the fibroblasts. A) Determination of the abundance of COL1A1 and αSMA in MRC‐5 cells upon TGF‐β1 and rCTSK (0.1 µg mL^−1^) stimulation in culture medium with glutamine (Gln^+^) or without glutamine (Gln^−^) for 0, 4, and 24 h. B) Determination of the abundance of COL1A1 and αSMA in MRC‐5 cells upon TGF‐β1 and rCTSK (0.1 µg mL^−1^) stimulation in culture medium with or without additional glutamine (2 mm) for 0, 4, and 24 h. C–E) Determination of the abundance of COL1A1 and αSMA in MRC‐5 cells upon TGF‐β1 and rCTSK (0.1 µg mL^−1^) stimulation in culture medium with GLS1 inhibitors including CB839 (10 µm) (C), BPTES (10 µm) (D), or DON (25 µm) (E) for 0, 4, and 24 h. F) Experimental diagram of BLM‐induced PF mice models with the addition of rCTSK intratracheally at Day 7, 9, and 11 respectively. The glutaminase inhibitor DON was injected at Days 8, 10, and 12 as well. Mice were sacrificed at Day 28 and subjected to further experiments. G) Determination of the abundance of COL1A1 and CTSK in the lung tissues from BLM‐induced PF mice with or without rCTSK and DON treatment. H) Determination of hydroxyproline contents in lung tissues from BLM‐induced PF mice with or without rCTSK and DON treatment by ELISA (n = 5 per group). I) Masson's staining of the lung tissues from BLM‐induced PF mice with or without rCTSK and DON treatment with statistical comparison. Scale bar, 1 mm (left) and 100 µm (right). J) Sirius red staining of lung tissues BLM‐induced PF mice with or without rCTSK and DON treatment with statistical comparison (left). Scale bar, 1 mm (left) and 100 µm (right). Data were presented as mean ± standard deviation (SD). *:*p* < 0.05, **:*p* < 0.01, ***:*p* < 0.001, NS, not statistically significant by the Student's *t*‐test. Unless indicated, data were representative of two independent experiments.

Since we observed elevated glutamate levels in the serum and cytosol upon rCTSK treatment, we determined whether glutamate was required for rCTSK‐associated COL1A1 overproduction. First, we stimulated MRC‐5 cells with TGF‐β1 and rCTSK in a glutamine‐free culture medium. rCTSK‐induced COL1A1 overproduction was dramatically reduced under glutamine‐free conditions, whereas α‐SMA levels in the cells remained comparable between the glutamine‐complemented and glutamine‐free culture media (**Figure** **6**A). When we cultured MRC‐5 cells in the medium with additional glutamine upon TGF‐β1 and rCTSK treatment, it was obvious that extra glutamine was sufficient to increase COL1A1 production in MRC‐5 fibroblasts (Figure 6B). Glutamine is converted into glutamate depending on GLS1 enzymatic activity.^[^ ^35^ ^]^ Therefore, we used the glutaminase inhibitors CB839, BPTES, and DON to determine the effect of GLS1 on rCTSK‐induced COL1A1 overproduction. As expected, treatment with the three inhibitors clearly reduced rCTSK‐promoted COL1A1 production, but not α‐SMA production, in MRC‐5 cells upon TGF‐β1 and rCTSK treatment (Figure 6C–E).

We used a GLS1 inhibitor, DON, to validate the involvement of glutamine metabolism in CTSK triggered COL1A1 overproduction in BLM‐induced PF mice. DON has been used in clinical trials for multiple tumors.^[^ ^36^, ^37^ ^]^ Its efficacy in modulating lung regeneration and the progression of PF has not yet been characterized. Therefore, we treated BLM‐treated mice with rCTSK or rCTSK in combination with DON (Figure 6F). We found that COL1A1 (Figure 6G) and hydroxyproline levels (Figure 6H) in the lung tissues were dramatically reduced in BLM‐induced PF mice treated with rCTSK and DON compared to mice treated with rCTSK alone. Collagen deposition by Masson's staining (Figure 6I) and fiber structures (Figure 6J) by Sirius Red staining of lung tissues also demonstrated attenuation of the PF progression with DON treatment. The mRNA expression of fibrosis markers was also inhibited (Figure S7, Supporting Information). Taken together, glutamine metabolism pathway has partially engaged in CTSK‐triggered collagen synthesis in the fibroblasts.

### 2.7 CTSK is Correlated with Glutamine Levels with a Poor Prognosis in Patients Suffering PF

**Figure 7:**
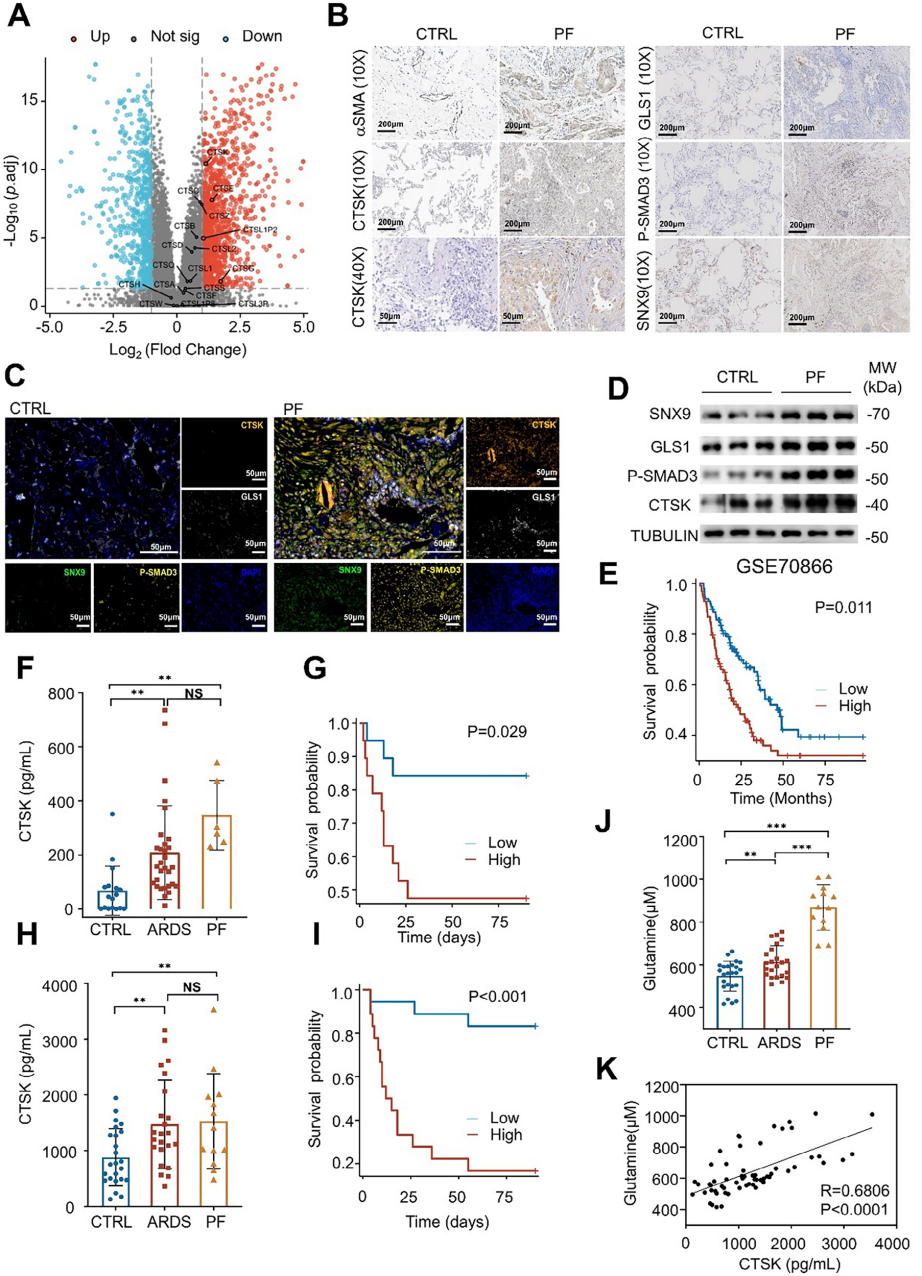
Peripheral CTSK content is associated with glutamine levels in acute respiratory distress syndrome (ARDS) patients bearing PF with a poor prognosis. A) Heatmap showing expression levels of 18 CTS family members in the lung tissues from IPF patients compared to normal control from the RNA‐seq dataset GSE124685. B) Determination of the abundance and intracellular localization of CTSK, SNX9, p‐SMAD3, and GLS1 in the healthy and PF lung tissues by immunohistochemical assay. αSMA serves as the positive control. Scale bar, 200 µm or 50 µm. C) Multiplex immunofluorescence staining of CTSK, SNX9, p‐SMAD3, and GLS1 in the healthy and PF lung tissues. Scale bar, 50 µm. D) Determination of the protein abundance of CTSK, SNX9, p‐SMAD3, and GLS1 in healthy and PF lung tissues by Western blotting. E) Kaplan‐Meier analysis of survival probability between CTSK highly and CTSK lowly expressing PF patients based on the GSE70866 dataset. F) Determination of CTSK concentration in BALF fluid from healthy donors, ARDS patients, and ARDS patients bearing fibrosis by ELISA. G) Kaplan‐Meier Analysis of the survival probability between the CTSK‐high and CTSK‐low ARDS patients. H) Determination of CTSK concentration in the serum from healthy donors, ARDS patients, and ARDS patients bearing fibrosis by ELISA. I) Kaplan‐Meier Analysis on the survival probability of ARDS patients based on peripheral CTSK contents. J) Determination of glutamine contents in the serum among the healthy donors, ARDS patients, and ARDS patients with fibrosis by ELISA. K) Correlation between serum CTSK and glutamine contents from healthy donors and ARDS patients by using the Spearman analysis. Data were presented as mean ± standard deviation (SD). *:*p* < 0.05, **:*p* < 0.01, ***:*p* < 0.001, NS, not statistically significant by the Student's *t*‐test.

Our in vitro and in vivo results from mouse models support CTSK as an extracellular ligand that interacts with cell membrane SNX9, which is involved in exaggerated collagen synthesis in fibroblasts. To determine the clinical relevance of these results, we analyzed published bulk RNA‐seq data from patients with PF (GSE124685) and found that the expression levels of CTS family members were upregulated in lung tissues from patients with PF, where *CTSK* exhibited the highest expression (**Figure** **7**A). Immunohistochemical analysis (Figure 7B) confirmed the upregulation of CTSK, SNX9, GLS1, and P‐SMAD3 in the lung tissues of patients with PF, while multiplex immunofluorescence revealed their colocalization with accompanying upregulation in fibrotic lung tissues (Figure 7C). Western blot analysis further validated their upregulation in the lung tissues of patients with PF (Figure 7D). These results indicate that CTSK, SNX9, GLS1, and P‐SMAD3 are coordinately upregulated and spatially co‐localized in fibrotic lung tissues, suggesting their potential synergistic roles in the pathogenesis of PF.

Excessive accumulation of CTSK is accompanied by PF progression in mouse models; therefore, the abundance of CTSK might be a potential predictive indicator of PF. By analyzing RNA‐seq data from patients with PF (GSE70866), we found that a high abundance of *CTSK* expression was significantly correlated with a low survival rate in patients with PF (Figure 7E). In this study, we collected BALF samples from 36 patients with pneumonia and ALI‐related ARDS and 17 healthy volunteers. Among 36 patients, six were diagnosed with PF using computed tomography (CT). Among patients with ARDS, those with PF exhibited a significantly higher abundance of CTSK in the BALF than those without PF, as well as CTSK levels in healthy volunteers (Figure 7F). Consistently, patients with a high abundance of CTSK showed an extremely high mortality rate compared to patients with a low abundance of CTSK (Figure 7G).

Several studies have reported that CTS family members can be detected in the plasma, and plasma CTSS has been recognized as an early indicator of liver fibrosis.^[^ ^18^ ^]^ Therefore, we measured serum CTSK levels in 24 healthy volunteers and 36 patients with pneumonia and ALI. Thirteen of the 36 patients with ALI were diagnosed with PF by CT. We again observed higher concentrations of CTSK in ALI patients with PF than in healthy volunteers (Figure 7H). A high mortality rate correlated with a high abundance of CTSK (Figure 7I). We also measured the glutamine levels in the sera of these patients. Similarly, serum glutamine levels were higher in patients with ARDS and PF than in healthy controls, and a more significant increase was observed in patients with PF (Figure 7J). More significantly, serum CTSK levels were positively correlated with glutamine levels (Figure 7K). These results from clinical PF samples indicate that CTSK accumulates in PF lung tissues as well as in the serum. Its strong correlation with serum glutamine levels makes it a prognostic indicator of PF.

## 3 Discussion

Early prediction of PF could facilitate proper clinical intervention to prevent the development of PF, which till now is not available due to poor understanding of the comprehensive processes of PF. Recently, the involvement of CTS family members in lung injury‐induced fibrosis has been addressed yet with controversial conclusions.^[^ ^38^ ^]^ Herein we have shown that CTSK was most abundantly expressed in the fibroblasts from PF mouse models as well as in human lung samples with PF, which is similar to silica‐induce PF mice.^[^ ^21^ ^]^ Moreover, we have elucidated that CTSK could serve as an extracellular ligand to interact with SNX9 followed by endocytosis and activation of TGF‐β1‐SMAD3 signaling for downstream collagen synthesis partially depending on glutamine metabolism. From our clinical data, we intend to demonstrate the feasibility of CTSK as well as glutamine in prognostic prediction of PF and developing new strategies for the prevention of PF.

Previous studies have revealed that in CTSK transgenic mice there exhibited significantly reduced PF at Day 7 when the fibrotic symptom was slight in BLM‐induced mice. However, on Day 14 PF aggravated with the accumulation of CTSK, indicating the complexity of CTSK in modulation of PF. Based on our study we have observed gradual increase in CTSK in the lung tissues from ALI and BLM‐induced PF mice. Although CTSK is beneficial for the degradation of ECM and facilitates lung regeneration, high abundance of CTSK makes it be an extracellular ligand to exacerbate collagen production in the fibroblasts and promotes the progression of PF. CTSK was previously reported to facilitate ECM production in the alveoli and interstitium,^[^ ^39^ ^]^ Our analysis on CTSK deficient mice confirmed the protective effects in alleviating PF progression by endogenous CTSK. However, excessive accumulation of CTSK in the pathological condition is more likely to promote collagen production for ECM accumulation and PF progression which was supported by scRNA‐seq data analysis and rCTSK‐treated mice model in this study. Therefore, our study innovatively elucidates the dual regulatory mechanism of CTSK in PF. Specifically, the moderate expression of CTSK in response to ALI in the early stage promotes extracellular matrix degradation through its enzymatic activity that restrains the PF progression, which also limits the accumulation of CTSK abundance for activation of excessive collagen production. While accompanied with disease progression of severe ALI, the continuous CTSK production derived from PF progression alternatively activates glutamine metabolism to promote collagen production, aggravating fibrosis and forming a vicious cycle for disease progression. This discovery provides new insights into the complex role of CTSK in PF and offers important theoretical foundations for CTSK‐targeted therapeutic strategies.

Considering that the serum concentration of CTSK abundance is correlated with the progression of ARDS patients, it would thus be reasonable to further evaluate serum CTSK abundance as the potential indicator for ALI and PF progression triggered by multiple insults with multi‐centric clinic trials. The identification of a robust range of serum CTSK abundance to predict whether ALI patients progress into PF or not would benefit the proper clinical intervention for improving the prognosis of ALI patients. Except the well‐established supportive clinical strategies for treating ALI and PF progression, recombinant CTSK peptides would be a valuable potency to directly target PF progression, however, should be precisely administrated determined by the serum concentration of CTSK abundance, which could be further evaluated in the future study.

The CTS family members, including CTSK, have been well characterized to undergo organelle transport as well as secret into the extracellular region for the degradation of ECM in multiple organs.^[^ ^40^ ^]^ Lysosomal CTSs are able to cleave the ectodomains of Toll‐like receptors 7 and 9 to participate innate immune response.^[^ ^41^ ^]^ CTSL and CTSD are required for autolysosomal degradation during autophagy while CTSS is involved in fusion processes of autophagosomes and lysosomes.^[^ ^42^ ^]^ CTSB modulates lysosomal calcium fluxes and represses TFEB transcriptional activation.^[^ ^43^ ^]^ Several other CTS members are involved in inflammation and nuclear translocation as well. These observations reveal intracellular trafficking of CTS family members to mediate cytosolic functions. In this study we found that extracellular CTSK was able to interact with SNX9 for endocytosis and facilitate TGF‐β1 induced SMAD3 activation, which in turn induced GLS1 expression and collagen synthesis, providing an alternative working model of CTSK in PF progression. SNX9 is a clathrin‐mediated endocytosis auxiliary protein that can influence different cellular processes by coordinating membrane transport and remodeling through changes in actin dynamics.^[^ ^44^ ^]^ It can promote nuclear translocation of SMAD3, a key transcription factor downstream of TGF‐β1 signaling pathway for PF.^[^ ^31^ ^]^ Interestingly, CTSK and several other CTS molecules have been shown to localize on nuclear membrane to modulate SMAD2/SMAD3 nuclear translocation.^[^ ^42^ ^]^ Our data have shown that cell membrane SNX9 could interact with extracellular CTSK to internalize CTSK and transport CTSK in the cytosol and nuclear membrane to mediate SMAD3 nuclear translocation and activation, which coordinates TGF‐β1‐triggered signaling pathway. Blockage of SNX9 activity suppressed the translocation of CTSK and downstream collagen synthesis, highlighting the roles of CTSK‐SNX9 interaction in mediating alternative mechanisms of CTSK in PF pathogenesis. Moreover, based on our mechanistic study, a combination of CTSK peptides with either glutaminase inhibitors or CME inhibitors for patients suffering severe PF would be worth to further examined in future studies.

Collagen is one of the major components of ECM that promotes PF. Proline is essential for the special triple helix structure formation of collagen.^[^ ^45^ ^]^ Amino acid metabolism, especially glutamine metabolism, is responsible for *de novo* synthesis of proline.^[^ ^35^, ^46^, ^47^ ^]^ Moreover, glutamine metabolism has been reported to upregulate during PF to meet high anabolic demands^[^ ^48^, ^49^, ^50^ ^]^ which facilitates the synthesis of proline and glycine. More specifically, proline biosynthesis can be either from direct metabolic conversion of glutamine or from the tricarboxylic acid cycle intermediate α‐ketoglutarate that can be converted from glutamine.^[^ ^33^ ^]^ Therefore, glutamine metabolism becomes an important pathway for the biosynthesis of proline from scratch.^[^ ^51^ ^]^ Our study reveals that CTSK association with SNX9 enhances TGF‐β1 induced SMAD3 activation and GLS1 expression. GLS1 then converts glutamine into glutamate and promotes subsequent glutamine metabolism and collagen production dedicating to PF progression. Our study thus emphasizes the importance of glutamine metabolism downstream of SMAD3 for collagen production activated by the CTSK‐SNX9 axis. Importantly, TGF‐β1‐activated SMAD3 has been extensively characterized as a key regulator of collagen expression and PF progression, exerting its effects through multiple downstream targets, including WNT/β‐catenin, PI3K/AKT, mTOR, and MAPK signaling pathways.^[^ ^52^, ^53^ ^]^ Therefore, further studies are required to evaluate the potency of these routes for pathological CTSK accumulation mediated PF progression.

The importance of CTSK in pathological lung diseases is not limited to ALI and PF progression. It has been reported that CTSK is also dramatically and specifically upregulated in patients’ lungs suffered Lymphangioleiomyomatosis (LAM) and LAM‐associated fibroblasts.^[^ ^54^ ^]^ Considering that the upregulated CTSK could be activated by mTOR signaling while mTOR inhibitor is the only proven treatment for LAM, the upregulated CTSK in LAM could potentially aggravated disease progression. Similarly, it has been reported that CTSK is also highly expressed in wide spectrum of perivascular epithelioid cell neoplasms (PEComas), including lung.^[^ ^55^ ^]^ Whether CTSK accumulation in LAM and PEComas would undergo endocytosis facilitated by SNX9 to activate specific intracellular signals for disease progression remains to be further elucidated.

**Figure 8:**
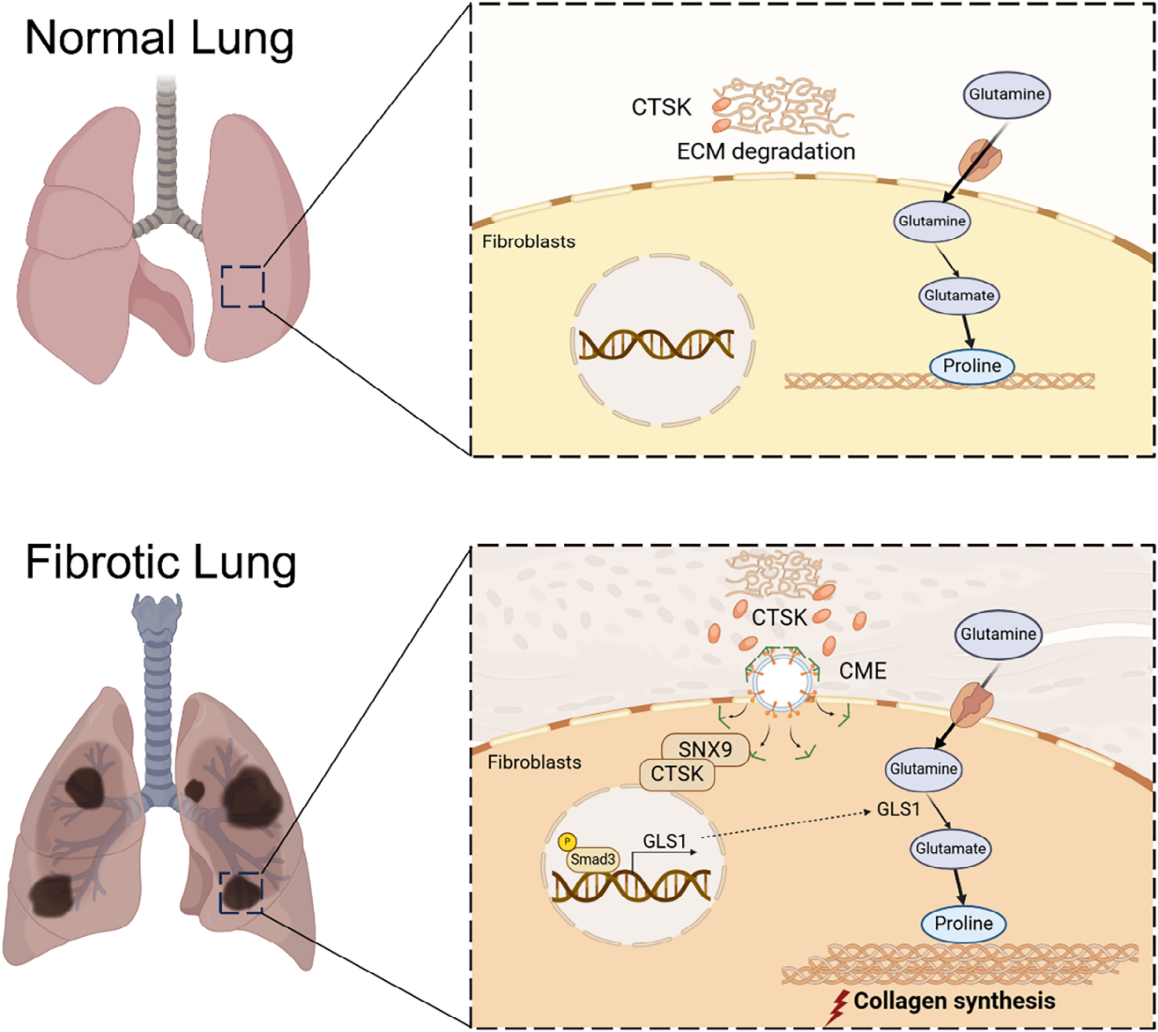
Schematic diagram.

In conclusion, we have demonstrated in this study that excessive accumulation of CTSK interacts with SNX9 to enhance TGF‐β1 induced SMAD3 activation and GLS1 expression in the fibroblasts, leading to the exacerbation of glutamine metabolic pathway for collagen biosynthesis and PF progression (**Figure** **8**). The abundance of serum CTSK and downstream glutamine may be a potential predictive biomarker of PF. Considering that certain glutamine metabolism inhibitors have been tested in phase II clinical trials for the treatment of other diseases, our study might provide new indications of glutamine metabolism inhibitors to treat PF in the future.

## 4 Experimental Section

### Human Samples

Six fibrotic lung tissues were collected from patients with IPF^[^ ^56^ ^]^ while control lung tissues from 6 noncancerous lung tissues were derived from patients with lung cancer who received treatment at Shanghai General Hospital and Wuxi People's Hospital. This study was approved by the ethical committees of Shanghai General Hospital (20211KY041 and 2 024 076) and Wuxi People's Hospital (KY24083). Before enrolment, all participants signed a written informed consent form. The study complied with the ethical guidelines of the 1975 Declaration of Helsinki.

### Animal Models

To establish a BLM‐induced PF mouse model, 8–10 weeks old male C57BL/6J mice were intratracheally injected once with BLM (3 mg kg^−1^; Selleckchem, Japan) or saline.^[^ ^57^ ^]^ After 28 days, the mice were euthanized in the presence of CO_2,_ and lung tissues were collected for further analysis. In some experiments, inhibitors have been used to investigate the pathogenic mechanisms. Mice were bred and housed at the Shanghai General Hospital Laboratory Animal Center. They were housed under specific pathogen‐free (SPF) conditions with free access to water and food. All procedures were performed according to protocols approved by the Committee for Animal Research of Shanghai Jiaotong University (2021AWS0114) and conformed to the Guide for the Care and Use of Laboratory Animals.

CTSK^fl/fl^ mice and Col1a2CreERT mice were purchased from Cyagen. Genetically targeted mice (Col1a2CreERT; CTSK^fl/fl^) were genotyped by standard PCR. To activate the Cre‐ERT system, the mice were intraperitoneally injected with tamoxifen (80 mg kg^−1^, Sigma‐Aldrich) every other day from days 7 to 21 after BLM treatment. Gene deletion efficiency was validated seven days after tamoxifen injection by PCR and qRT‐PCR using tissue‐specific primers.

### Cell Culture

The MRC‐5 human fibroblast cell line was obtained from the Chinese Academy of Sciences Cell Bank (Shanghai, China), and cultured in Dulbecco's modified Eagle's medium (Gibco, USA) containing 10% fetal bovine serum (FBS) (Gibco, USA) at 37 °C and 5% CO_2_ atmosphere. To mimic the fibrosis in vitro, MRC‐5 cells were cultured in DMEM culture medium containing transforming growth factor‐β1 (TGF‐β1) (PeproTech, Rocky Hill, NJ, USA) for 24 h^[^ ^58^ ^]^ and collected for further experiments.

### RNA Extraction and Quantitative Real‐Time Polymerase Chain Reaction (qRT‒PCR)

RNA‐easy Isolation Reagent (Vazyme, Nanjing, China) was used to extract total RNA from the tissue or cell samples. The concentration and purity of the total RNA were determined and reverse‐transcribed to cDNA using a HiScript III RT SuperMix kit (Vazyme, Nanjing, China). qRT‐PCR was performed using the qPCR SYBR Green Master Mix (Vazyme, Nanjing, China). The primers (Sangon Biotech, China) are listed in the Supplementary Table. Data were normalized to GAPDH and the expression levels of target genes were calculated by 2^[‐ (Ct target gene‐Ct GAPDH)]^.

### Western Blot

Western blot analysis was performed as previously described.^[^ ^56^ ^]^ Briefly, cells or tissues were harvested and lysed in RIPA lysis buffer (Beyotime, China). The protein concentrations of the extracted samples were measured using a BCA protein assay (Thermo Fisher Scientific, USA). Equivalent amounts of protein (25–40 µg) were separated by 7.5% or 10% SDS‐PAGE and transferred to a nitrocellulose membrane. The membrane was blotted in 5% skim milk in Tris‐buffered saline with tween‐20 (TBST, pH7.4) for 2 h at room temperature (RT) and was incubated with primary antibody overnight at 4 °C, followed by incubation with horseradish peroxidase‐conjugated secondary antibodies for 1 h at RT. Target proteins were detected using a chemiluminescence detection system (Thermo Fisher Scientific, USA). Antibody information used in this study is listed in Supplementary Table S1 (Supporting Information).

### Immunoprecipitation

To investigate the association between SNX9 and CTSK, TGF‐β1‐treated MRC‐5 cells were stimulated with CTSK‐HIS and lysed in RIPA buffer (Beyotime) supplemented with protease inhibitors. The lysates were quantified and incubated with an anti‐CTSK antibody (1 µL per sample) overnight, followed by the incubation with 10 µL protein A/G beads (Santa Cruz, SC‐2003) for another 3 h at 4 °C. The immune complexes were washed thrice with PBS and subjected to Western Blot analysis. Detection antibodies used in this study were listed in Supplementary Table S1 (Supporting Information).

### Protein Interaction Prediction and Mutant Design

The interaction interface between CTSK and SNX9 was predicted using the AlphaFold3. Of these, the section of focus is the SH3 region of SNX9. Based on the predicted binding sites, two critical amino acid residues in each protein were selected for alanine‐screening mutagenesis. Point mutants were designed as follows: CTSK: [Mutations 1 (K153A, K154A, K155A) and 2 (K220A, C221A)], SNX9 [Mutations 3 (N17A and E19A) and 4 (W39A)]. Mutant constructs were generated via homologous recombination and cloned into the pLVX plasmid vector. The resulting plasmids were verified using Sanger sequencing to ensure the correct introduction of mutations. To validate the predicted interactions, MRC5 cells were transiently transfected with wild‐type or mutant plasmids encoding CTSK and SNX9. After 24 h of transfection, cells were stimulated with 10 ng mL^−1^ TGF‐β1. The cells were lysed after 24 h. The lysates were quantified and incubated with an anti‐FLAG antibody or anti‐MYC antibody (1 µL per sample) overnight, followed by the incubation with 10 µL protein A/G beads (Santa Cruz, SC‐2003) for another 3 h at 4 °C. The precipitated proteins were analyzed by western blotting using anti‐FLAG and anti‐MYC antibodies. The immune complexes were washed thrice with PBS and subjected to Western blot analysis. Antibodies used in this study are listed in Supplementary Table S1 (Supporting Information).

### AFM

Tissue stiffness assays were performed in the contact mode using a FastScan Bio Fast AFM (Bruker, USA). Force spectra were plotted using a spherical colloidal probe (MLCT‐O10, Bruker, USA) with a nominal spring constant of 0.1 N m^−1^ and a tip radius of 6 µm. The spring constant was calibrated using the thermal noise method. All force curves were analyzed using the Hertzian contact model in Nanoscope Analysis software (Bruker, USA). A Poisson's ratio of 0.5 was assumed for the samples, and Young's modulus was calculated from the indentation data. All experiments were performed at room temperature (25 ± 1 °C) and relative humidity (50 ± 5%). Each sample was measured within 1 h of preparation to minimize potential changes in sample properties. More than ten force diagrams were collected for each sample, and 30 different samples were analyzed for each condition. Statistical significance was analyzed using one‐way ANOVA, with *p* < 0.05 considered significant.

### Histology

Fresh lung tissues from mice or patients were fixed overnight in 4% paraformaldehyde (PFA) for dehydration, embedded in paraffin, and sectioned into 5 µm thickness. Immunohistochemical staining was performed routinely to observe histopathological changes in lung tissues using a light microscope (×100, ×200). Sirius Red (Abcam, Cambridge, UK) and Masson's trichrome staining (Sigma Aldrich, MO, USA) were used to detect collagen fibers and collagen deposition, respectively, in lung tissue sections. The reagents used are listed in Supplementary Table S1 (Supporting Information).

### Multiplex Immunofluorescence Staining

Frozen tissue samples were embedded in OCT and sectioned into 5 µm‐thick slices. Slides were fixed in 4% paraformaldehyde for 15 min and subjected to antigen retrieval in 10 mm sodium citrate buffer (pH 6.0) at 95 °C for 20 min. Nonspecific binding was blocked with 5% BSA for 1 h. Primary antibodies against CTSK (1:50), SNX9 (1:200), GLS1 (1:200), and p‐SMAD3 (1:200) were sequentially applied and the membranes were incubated for 1 h at room temperature. Fluorophore‐conjugated secondary antibodies (Cy5, SpGreen, Aqua, and SpOrange) were sequentially applied and incubated in the dark for 1 h at room temperature. Nuclei were counterstained with DAPI (1 µg mL^−1^) for 10 min. Slides were mounted with an anti‐fade mounting medium and imaged using a laser scanning confocal microscope (Leica TCS SP8, Leica). Antibodies used in this study are listed in Supplementary Table S1 (Supporting Information).

### Serum Collection from Mice

Blood samples were collected from the mice via orbital sinus puncture under anesthesia. The mice were anesthetized by intraperitoneal injection of pentobarbital sodium (50 mg kg^−1^ body weight) to minimize discomfort. The orbital area was gently massaged to promote blood flow, and a sterile glass capillary tube was inserted into the medial canthus of the eye at an angle of 30–45°. Blood was allowed to flow into the capillary tube by capillary action and collected in a 1.5 mL microcentrifuge tube. After collection, pressure was applied to the orbital area using a sterile cotton swab to ensure hemostasis. Blood samples were left at room temperature for 30 min to allow clotting, followed by centrifugation at 2000 × g for 15 min at 4 °C to separate serum. The supernatant (serum) was carefully transferred to a new microcentrifuge tube and stored at −80 °C until further analysis.

### Immunofluorescence Assay

MRC‐5 cells were fixed with 4% PFA for 10 min, permeabilized with 0.1% TritonX‐100 for 6 min, and blotted with PBS containing 0.2% BSA for 30 min at RT. Primary antibodies were added and the cells were incubated for 1 h at RT. After washing three times with PBS, the cells were incubated with Alexa Fluor‐conjugated goat anti‐mouse secondary antibody (Thermo, A32723, 1:2000) or HRP‐conjugated goat anti‐rabbit secondary antibody (Bioss, bs‐0295G, 1:500) for 1 h at RT. DAPI was added 5 min before image scanning using a laser‐scanning confocal microscope (Leica TCS SP8, Leica).

### Lentivirus Preparation and Cell Infection

Puromycin‐resistant pLKO.1 lentiviral vector containing shRNA targeting *SNX9* was constructed. HEK293T cells were plated in 6 cm dishes and transfected with 1.5 µg of pLKO.1‐*shSNX9* together with 1.5 µg of packaging plasmid (psPAX2) and 1.5 µg of envelope plasmid (pMD2.G) using Lipofectamine 3000 (Invitrogen, USA). Lentivirus‐containing supernatants were collected after 24 h. MRC‐5 cells were infected using 2 mL of supernatant mixed with 1.5 mL of DMEM containing 10% FBS and 5 µg mL^−1^ polybrene for 24 h. Infected cells were cultured in DMEM culture medium containing 2.5 µg mL^−1^ puromycin for 2 weeks to generate stably‐expressing MRC‐5 cell lines. The shRNA sequences are listed in Supplementary Table S1 (Supporting Information).

### Isolation of Mouse Primary Fibroblasts from Lung Tissues

Mice were anesthetized with an intraperitoneal injection of pentobarbital sodium (50 mg kg^−1^ body weight) and disinfected with 70% ethanol applied to the thoracic area. The thoracic cavity was incised using sterile surgical scissors and the left lung lobe was carefully removed and placed in a sterile Petri dish containing ice‐cold PBS. The lung tissues were rinsed with sterile PBS, minced into 1 mm^3^ pieces using sterile scissors, and incubated in DMEM medium supplemented with 0.1% collagenase type I (Sigma‐Aldrich, USA) at 37 °C for 1 h with gentle agitation to facilitate enzymatic digestion. After digestion, the tissue fragments were washed thrice with PBS to remove residual collagenase. The cell suspension was centrifuged at 1500 × g for 10 min, and the pellet was resuspended in DMEM supplemented with 15% FBS (Gibco, USA) and 1% penicillin‐streptomycin (Gibco, USA). The cells were then seeded into 10 cm culture dishes and incubated at 37 °C in a humidified atmosphere with 5% CO₂. After 4‒5 days of culture, adherent fibroblasts were harvested by trypsinization using 0.25% trypsin‐EDTA (Gibco) and either passaged or used in further experiments.

### Hydroxyproline Assay

The hydroxyproline content in mouse lung tissues was determined using a hydroxyproline colorimetric assay kit (Nanjing Jiancheng, China) according to the manufacturer's protocol. Briefly, lung tissues were weighed, hydrolyzed in an alkaline solution, and mixed with the detection buffer. Supernatants were collected and incubated in a water bath before measuring the absorbance at 550 nm using a microplate reader.

### Determination of Glutamine Content

Glutamine content was determined using a Glutamine Assay Kit (Abcam, Cambridge, USA) according to the manufacturer's protocol. The amount of glutamine was calculated by measuring the amount of ammonia, based on the conversion of glutamine to glutamate and ammonia.

### Determination of Glutaminase Activity

Glutaminase (GLS) activity was measured using the GLS Activity Assay Kit (Sangon, China) according to the manufacturer's protocol. The enzyme activity was calculated by measuring the amount of ammonia produced from glutamine catalyzed by GLS using an indophenol blue colorimetric method.

### Extraction of Nuclear and Cytoplasmic Proteins

Nucleoplasm separation was performed using NE‐PER Nuclear and Cytoplasmic Extraction Reagents (Beyotime, China), following the manufacturer's protocol. Low osmotic pressure was used to sufficiently swell the cells and disrupt the cell membrane to release cytoplasmic proteins, followed by centrifugation to obtain nuclear precipitates. Cytosolic proteins were extracted using high‐salt cytosolic protein extraction reagent.

### Enzyme‐Linked Immunosorbent Assay

Bronchoalveolar lavage fluid (BALF) from patients and healthy volunteers was centrifuged at 3000 g for 8 min, and serum from patients and healthy volunteers was collected from fresh blood by centrifugation at 3000 g for 15 min. Mouse serum was collected as previously described. The BALF supernatants and serum were subjected to enzyme‐linked immunosorbent assay (ELISA) for the detection of CTSK concentration using commercially available ELISA Kits (Novus Biologicals, USA and Byabscience, China) according to the manufacturer's recommendations.

### Microarray Data Acquisition

We downloaded the transcriptome data (GSE124685 and GSE70866) from the Gene Expression Omnibus (GEO) database (https://www.ncbi.nlm.nih.gov/geo/). The GSE124685 dataset contained 95 lung tissue samples from 10 IPF patients and 6 healthy donors based on GPL17303 platform (Ion Torrent Proton, Homo sapiens).^[^ ^59^ ^]^ The GSE70866 dataset we used in this study contained 232 BALF samples from 212 IPF patients and 20 normal volunteers based on GPL14550 platform (Agilent‐028004 SurePrint G3 Human GE 8×60K Microarray) and GPL17077 platform (Agilent‐039494 SurePrint G3 Human GE v2 8×60K Microarray).^[^ ^60^ ^]^ Samples missing overall survival (OS) data were excluded. We used the limma package in R software to remove the missing probe values and took the median value to represent genes with multiple probes.

### ScRNA‐Seq Data Processing

ScRNA‐Seq) dataset of IPF was obtained from the GEO database (GSE111664, GSE136831, and GSE132771) that were reported by Aran et al.,^[^ ^61^ ^]^ Adams et al.,^[^ ^62^ ^]^ and Tsukui et al.^[^ ^25^ ^]^ Information on the construction of the mice model and the preparation of single cell samples was described in the original paper.^[^ ^25^, ^61^, ^62^ ^]^ GSE111664 dataset consisted of 6 lung samples from saline‐treated mice and 3 lung samples from BLM‐treated mice. GSE136831 dataset consisted of 32 lung samples from IPF, 28 lung samples from the smoker and nonsmoker. GSE132771 dataset consisted of two lung samples from Col1a1‐GFP reporter mice treated with BLM after 14 days. The following steps were performed for scRNA‐seq data processing routinely: a) quality control of preliminary data was carried out based on percent. Mito and nFeatures of each sample; b) samples were integrated using the merge function of Seurat (v4.0.5) followed by data standardization; c) Harmony (v0.1.0) was used for reclustering; and d) the cells were manually annotated using specific gene signatures or automatically annotated with SingleR (v1.8.1) after clustering by UMAP or tSNE dimensional reduction.^[^ ^61^ ^]^

### Bioinformatic Evaluation of Metabolic Activity

scMetabolism algorithm was used for single‐cell metabolic analysis and single‐sample gene set enrichment analysis (ssGSEA) was used to compare metabolic profiles between different cells in GSE136831. Metabolite abundance was deduced by applying single‐cell flux estimation analysis (scFEA) based on scRNA‐seq data.^[^ ^63^ ^]^ Gene set variation analysis (GSVA) was used to calculate enrichment scores for each metabolic pathway in the transcriptome data for each sample.

### Metabolomic Analysis: LC‐MS/MS Untargeted Metabolomic Analysis

Cells were spiked with 500 µL solution (methanol: water = 4:1, V/V) containing the internal standard and vortexed for 3 min. The samples were placed in liquid nitrogen for 5 min and on dry ice for 5 min, and then thawed on ice and vortexed for 2 min. This freeze‐thaw circle was repeated three times in total. The samples were then centrifuged at 12 000 rpm for 10 min (4 °C). A 300 µL of supernatant was collected and placed at −20 °C for 30 min. The sample was then centrifuged at 12 000 rpm for 3 min (4 °C). A 200 µL aliquots of supernatant were transferred for liquid chromatography‐tandem mass spectrometry (LC‐MS/MS) analysis. The sample extracts were analyzed using an LC‐ESI‐MS/MS system (UPLC, ExionLC AD, https://sciex.com.cn/; MS, QTRAP System, https://sciex.com/). The Triple TOF mass spectrometer was used to acquire MS/MS spectra on an information‐dependent basis (IDA). In this mode, the acquisition software (TripleTOF 6600, AB SCIEX) continuously evaluated the full scan survey MS data as it collected and triggered the acquisition of MS/MS spectra depending on preselected criteria. Obtaining LIT and Triple Quadrupole (QQQ) Scans on a Triple Quadrupole Linear Ion Trap Mass Spectrometer (QTRAP) for QTRAP LC‐MS/MS Systems. For two‐group analysis, differential metabolites were determined by VIP (VIP > 1) and *p*‐value (*p*‐value < 0.05, Student's *t*‐test). VIP values were extracted from OPLS‐DA results, which also contained score plots and permutation plots, and generated using R package MetaboAnalystR. The data were log‐transformed (log2) and mean centering before OPLS‐DA. In order to avoid overfitting, a permutation test (200 permutations) was performed.

### Metabolomic Analysis:GC‐MS/MS Targeted Amino Acid Metabolomics

The target metabolites were qualitatively and quantitatively analyzed using a Thermo scientific TSQ9000 gas chromatograph (GC) as the analytical instrument. Metabolite quantification was performed using triple quadrupole MS in multiple reaction monitoring (MRM) modes. The reproducibility of metabolite assays was determined by overlay display analysis of the total ion current plots (TIC plots) analyzed by MS detection of different quality control specimens, and the effect of technical repetition. Based on the information of retention time and peak shape of the metabolites, the MS peaks of each metabolite detected in different samples were manually corrected to ensure the accuracy of qualitative and quantitative analysis. The peak area of each chromatographic peak represented the relative contents of the corresponding metabolite. The mixed standard solution was prepared, and the gradient dilution of the mixed standard was carried out to obtain the corresponding quantitative MS data of the standard at different concentrations. The standard curves of different metabolites were plotted with the concentration of the standard (ng mL^−1^) as the horizontal coordinate and the peak area of the mass spectrometry peaks as the vertical coordinate. The peak areas of the metabolites were brought into the regression equations fitted from the standard curves to obtain the concentration information of the metabolites. According to the parameters of sampling and/or dilution, the metabolite concentration information was further calculated to obtain the absolute content of each metabolite. The quantitative information of all metabolites was statistically analyzed to obtain the mean value of each group of metabolites as well as the FC and *p*‐value values between the comparison groups.

### Metabolomic Analysis:LC‐MS/MS Protein Identification

Samples were thawed and repeatedly washed with PBS for 5 times. 50 mm NH_4_HCO_3_ solution containing 8 m urea was utilized to denature the protein samples, followed by the addition of 500 mm DTT aqueous solution to reach the final concentration at 10 mm. The samples were then mixed by slightly blowing, and water bathed at 55 °C for 30 min. After cooling down to RT, 15 mm iodoacetamide aqueous solution was added with slight blowing and mixing, followed by avoiding light reaction for 30 min. The samples were then added with 25 mm NH_4_HCO_3_ aqueous solution to make the concentration of urea below 1 m. The samples were then water bathed with 0.4 µg trypsin at 37 °C overnight, and centrifuged at 12 000 g for 5 min to collect the supernatant for evaporation. Next, peptide desalting was performed. Briefly, the volatilized peptide sample was re‐dissolved in Nano‐HPLC Buffer A, followed by activation, equilibration, peptide fixation, desalting, and elution. Finally, 80 µL of eluent phase Buffer B containing the peptide sample after desalting was volatilized and ready for further analysis by using a liquid‐mass spectrometry system consisting of an Ultimate 3000 nano ultra‐performance liquid tandem Q Exactive plus high‐resolution mass spectrometer. The volatilized peptide samples were re‐dissolved in Nano‐HPLC Buffer A, separated in a Nano‐HPLC liquid system UltiMate 3000 RSLCnano (ThermoFisher Scientific, USA) with a 75 µm × 150 mm analysis column (RP‐C18, New Objective, USA) at a flow rate of 300 nL/min, and cleaned up once with a 30 min mobile phase gradient of blank solvent. The enzymatic products were then separated by capillary high‐performance liquid chromatography (HPLC) and analyzed by MS using a Q‐Exactive plus mass spectrometer (ThermoFisher Scientific). In combination with the library search software ProteomeDiscover 2.5, samples from different groups were analyzed for protein qualitative (or relative quantitative) analysis. The list of proteins obtained in the experiment reflected the types and relative contents of proteins detected by this assay.

### Metabolomic Analysis: KEGG Annotation and Enrichment Analysis

Identified metabolites were annotated using KEGG Compound database (http://www.kegg.jp/kegg/compound/). Annotated metabolites were mapped to KEGG Pathway database (http://www.kegg.jp/kegg/pathway.html). Pathways with significantly regulated metabolites mapped to were then fed into MSEA (metabolite sets enrichment analysis). The significance was determined by hypergeometric test's p‐values.

### Statistical Analysis

Data were presented as the means ± standard deviation (SD). Statistical analyses included a two‐tailed unpaired Student's *t*‐test to compare the difference between paired groups and a one‐way analysis of variance (ANOVA) with a Bonferroni post‐test to assess the significance across the groups. Statistical significance was considered at a *p*‐value of <0.05.

## Conflict of Interest

The authors declare no conflict of interest.

## Author Contributions

M.C., X.M., Y.Z., and D.W. contributed equally to this work. Z.Yang. and R.W. generated the concept, designed the project and experiments, interpreted the results; M.C. designed and conducted the key experiments analyzed the data, and wrote the manuscript; X.M., Y.Z., M.W., and X.T. performed the experiments; Z.Yang., X.M., and R.W. supervised the study; P.W. provided clinical samples; Z.Yue., Z.W., and J.Z. discussed and interpreted the results. All of the authors approved the final manuscript.

## Supporting information



Supporting Information

## Data Availability

The data that support the findings of this study are available in the supplementary material of this article.
