# Supplementary material for: Cathepsin K Aggravates Pulmonary Fibrosis Through Promoting Fibroblast Glutamine Metabolism and Collagen Synthesis
Source: Adv Sci (Weinh). 2025 Jul 3;12(34):e13017. doi: 10.1002/advs.202413017 (PMC12442634; doi:10.1002/advs.202413017)
Supplement: Supplementary file 1 — Supporting Information [file ADVS-12-e13017-s001.docx]

**Supplemental Figures**
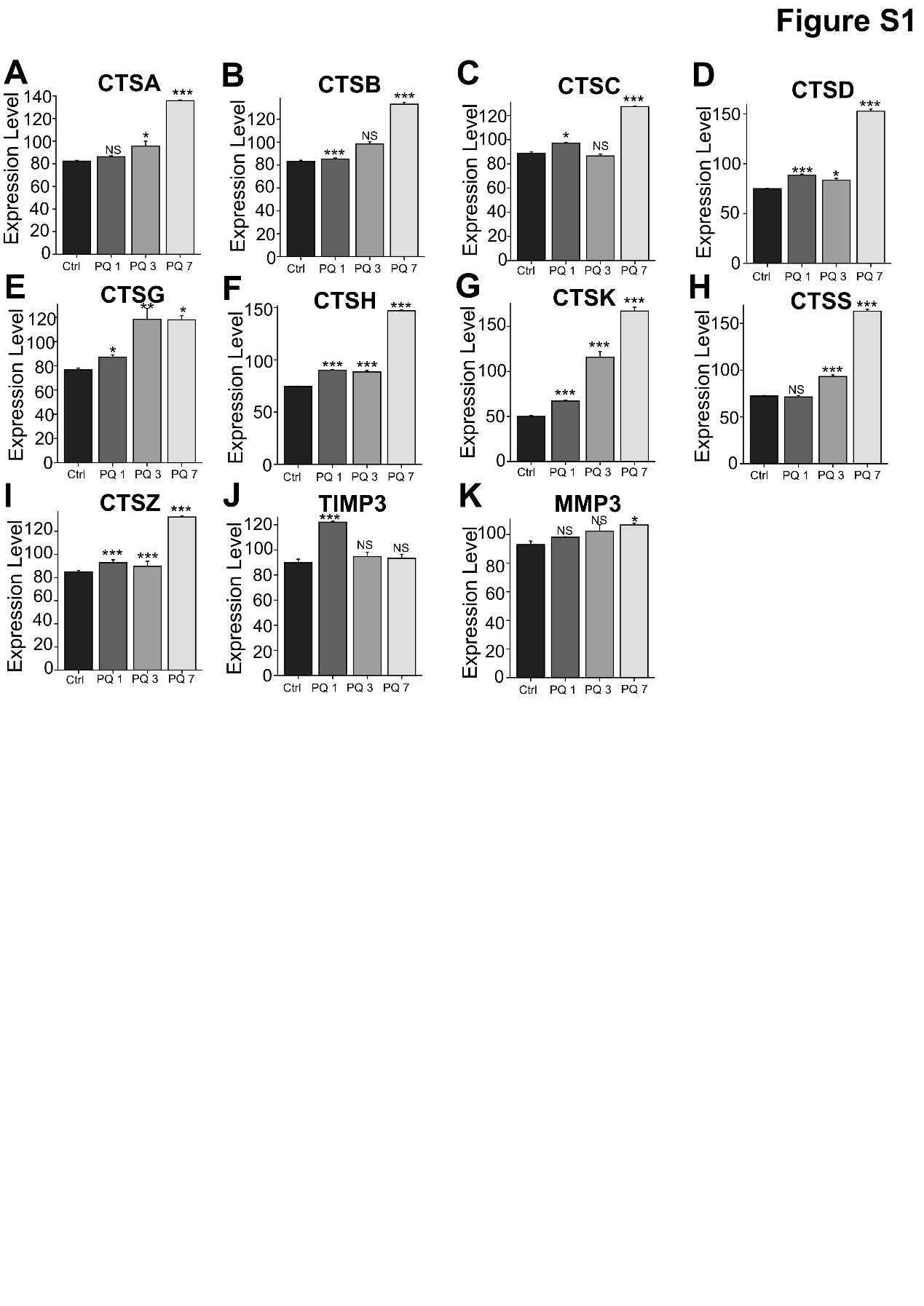


**Figure S1. Detection of the abundance of Cathepsin (CTS) family members in lung tissues from paraquat (PQ)-induced acute lung injury (ALI) mice models at Day1 (PQ1), Day3 (PQ3) and Day7 (PQ7), including CTSA (A), CTSB (B), CTSC (C), CTSD (D), CTSG (E), CTSH (F), CTSK (G), CTSS (H), CTSZ (I),TIMP3 (J) and MMP3 (K) by proteomic analysis.**

The data were presented as mean ± standard deviation (S.D.). *: *p* < 0.05, **:*p* < 0.01, ***: *p* < 0.001, NS, not statistically significant. n=3.


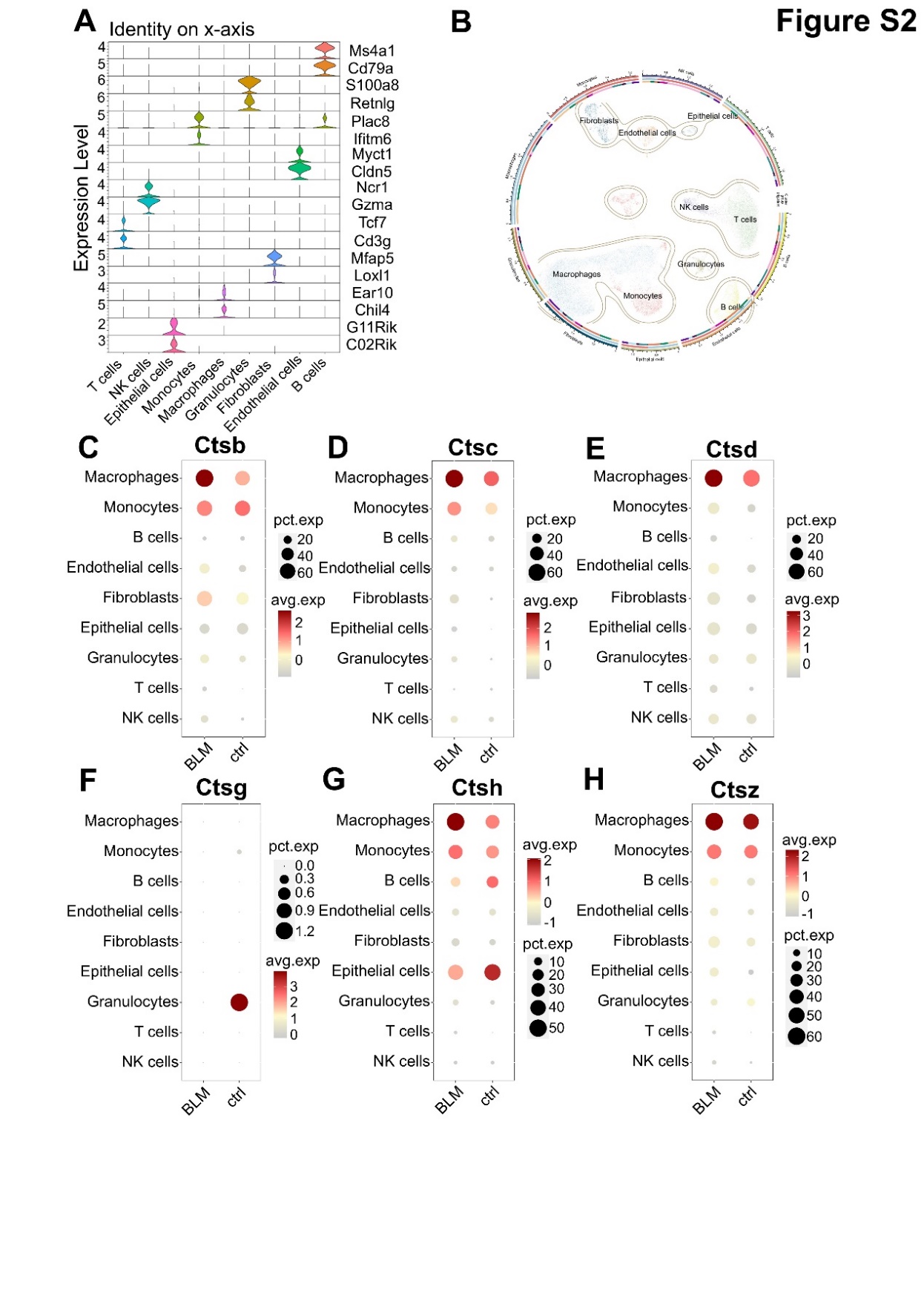


**Figure S2. Cathepsin gene expression profiles in the lung tissues from bleomycin-induced mice.**

**(A-B)** Single-cell sequencing data of the lung tissues from BLM-induced mice GSE111664 to define cell types by top2 gene signatures by using violin plot (A) and UMAP plot (B), including T cells, NK cells, B cells, epithelial cells, monocytes, macrophages, granulocytes, fibroblasts and endothelial cells.

**(C-H)** The expression profiles of *Ctsb* (C), *Ctsc* (D), *Ctsd* (E), *Ctsg* (F), *Ctsh* (G), *Ctsz* (H) in 9 cell types in the lung tissues of ctrl and BLM-induced mice based on sc-RNA sequencing data GSE111664 by using Bubble plot.


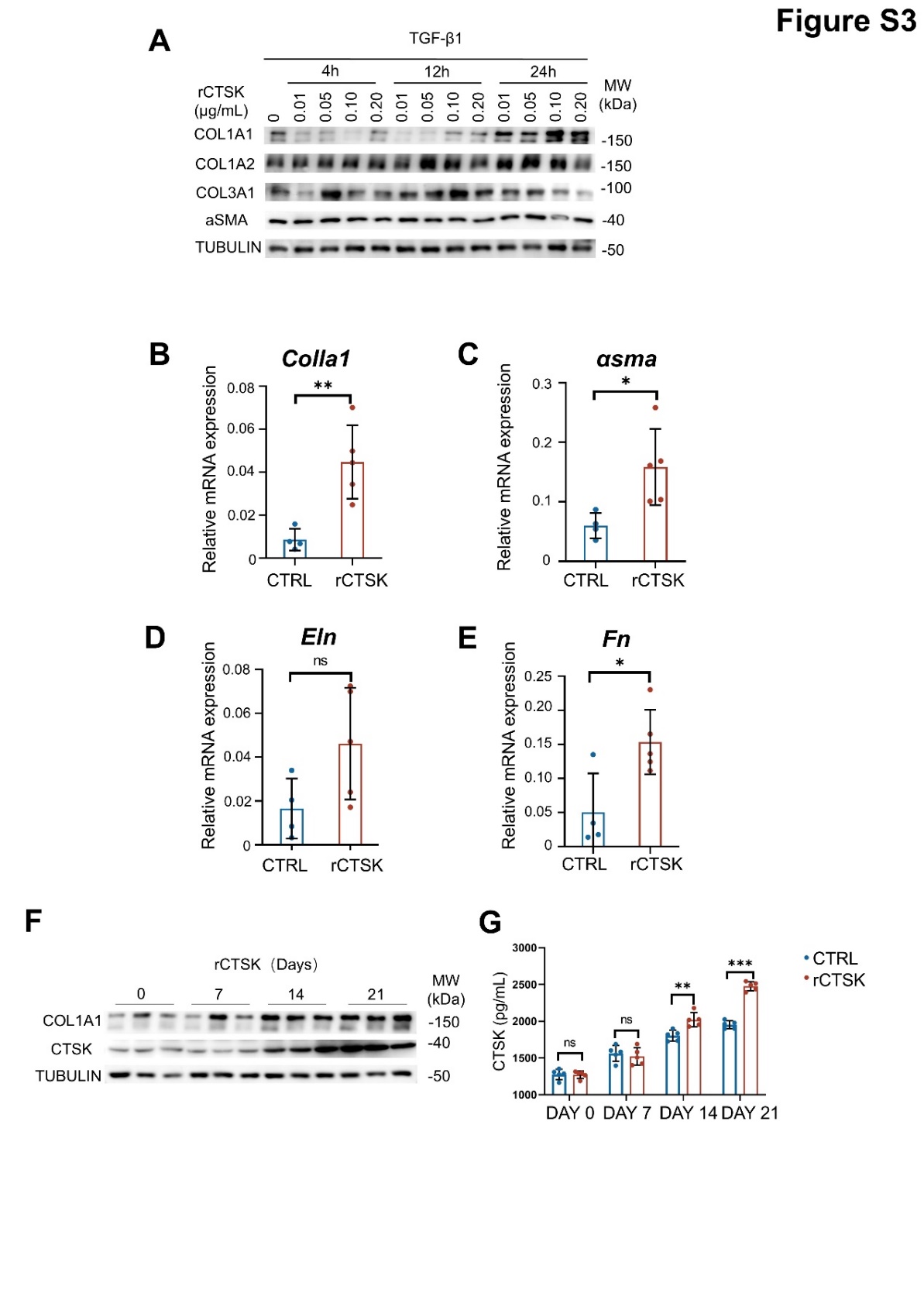


**Figure S3. CTSK aggravates the severity of PF.**

**(A)** Detection of expression levels of COL1A1, COL1A2, COL3A1 and αSMA in BLM-induced PF mouse primary lung fibroblasts followed by the addition of 0.02, 0.05, 0.1, 0.2 μg/mL rCTSK for 4, 12 and 24 hrs respectively by Western blotting.

**(B-E)** Expression levels of *Col1a1* (**A**), *αsma* (**B**), *Eln* (**C**) and *F*n (**D**) in the lung tissues from CTRL and rCTSK treated mice after BLM exposure. (n = 5 per group).

The data were presented as Mean ± s.d., **p*< 0.05, ***p*< 0.01, ****p* < 0.001, NS, not statistically significant.

**(F)** The expression levels of COL1A1 and CTSK in lung tissues of rCTSK-treated mice were detected by Western blotting after days 0, 7, 14, and 21 of BLM exposure. (n = 5 per group)

**(G)** The abundance of CTSK in mouse serum from CTRL and rCTSK-treated mice were detected by ELISA after days 0, 7, 14, and 21 of BLM exposure. (n = 5 per group)

**
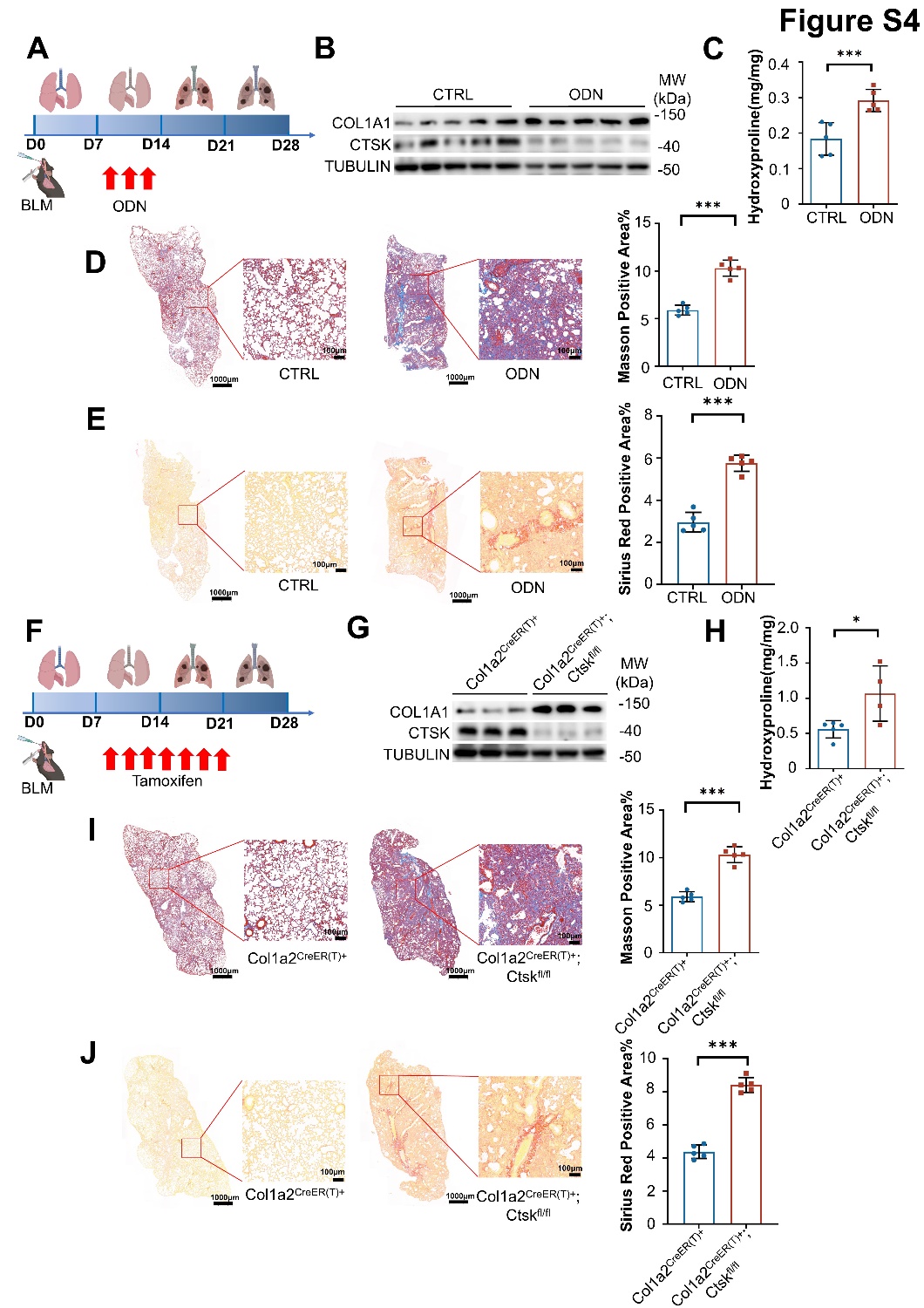
**

**Figure S4. Pathological significance of inhibition of CTSK activity and lung fibroblast-specific knockout of CTSK in PF in BLM mice.**

**(A)** Experimental diagram of inhibiting CTSK activity in BLM-induced PF mice model. Mice were sacrificed at Day 28 and subjected to further experiments. ODN, odanacatib.

**(B)** Expression levels of COL1A1 and CTSK in lung tissues between CTRL and ODN treated mice after BLM exposure by Western blotting.

**(C)** Comparisons of hydroxyproline contents in lung tissues between CTRL and ODN treated mice after BLM exposure by ELISA. Mean ± s.d., ****p*< 0.001, n = 5.

**(D)** Masson’s staining of the lung tissues in CTRL (left) and ODN (middle) treated-mice after BLM exposure with statistical comparisons of the percentages of Masson positive areas (right) (n = 5 per group). Scale bar: 1 mm and 100 μm respectively. ****p*< 0.001, n = 5.

**(E)** Sirius red staining of the lung tissues in CTRL (left) and ODN (middle) treated mice after BLM exposure with statistical comparison of the percentages of Sirius red positive areas (right) (n = 5 per group). Scale bar: 1 mm and 100 μm respectively. ****p*< 0.001, n = 5.

**(F)** Experimental diagram of conditional knocking out CTSK in fibroblasts of BLM-induced mice. Mice were sacrificed at Day 28 and subjected to further experiments.

**(G)** Expression levels of COL1A1 and CTSK in lung tissues between CTRL(Ctsk^fl/fl^) and Ctsk-cKO (Col1a2^CreERT+^; Ctsk^fl/fl^) mice after BLM exposure by Western blotting.

**(H)** Comparisons of hydroxyproline contents in lung tissues between CTRL(Ctsk^fl/fl^) and Ctsk-cKO (Col1a2^CreERT+^; Ctsk^fl/fl^) mice after BLM exposure by ELISA. Mean ± s.d., **p*< 0.05, n = 5.

**(I)** Masson’s staining of the lung tissues in CTRL (left) and Ctsk-cKO (middle) treated-mice after BLM exposure with statistical comparisons of the percentages of Masson positive areas (right) (n = 5 per group). Scale bar: 1 mm and 100 μm respectively. ****p*< 0.001, n = 5.

**(J)** Sirius red staining of the lung tissues in CTRL (left) and Ctsk-cKO (middle) treated mice after BLM exposure with statistical comparison of the percentages of Sirius red positive areas (right) (n = 5 per group). Scale bar: 1 mm and 100 μm respectively. ****p*< 0.001, n = 5.


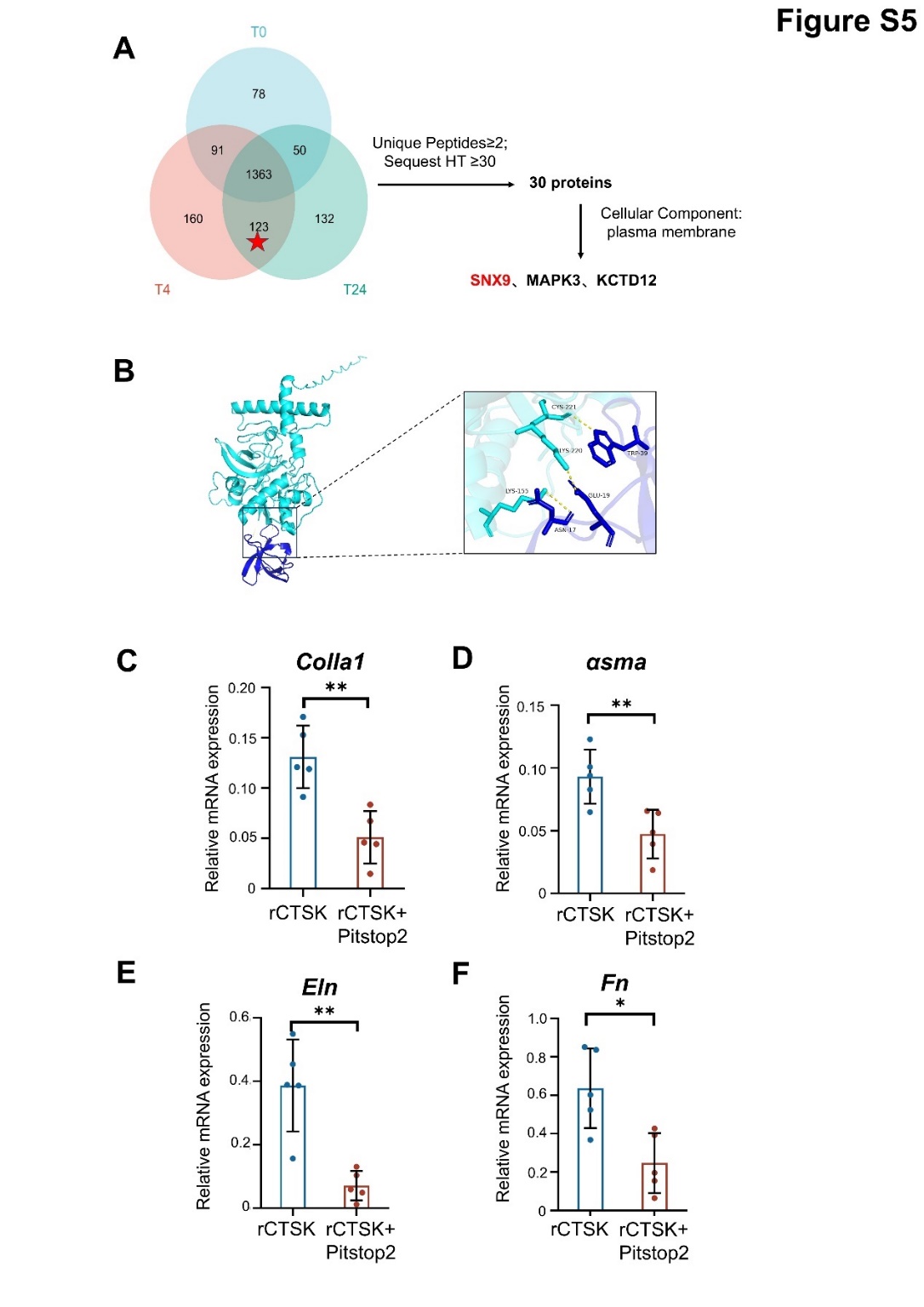


**Figure S5. SNX9 mediates CTSK endocytosis to promote collagen synthesis.**

**(A)** Schematic diagram showing the screening of CTSK-interacting proteins based on LC-MS/MS mass spectrometry assay. MRC-5 cells were treated with TGF-β1 and rCTSK (0.1 μg/mL) for 0, 4 or 24 hrs. Cell samples were collected, homeogenized and subject to LC-MS/MS mass spectrometry assays. Those who were absent in the ctrl group, enriched (unique peptides ≥2; sequest HT ≥30) in both T4 and T24 group with cell membrane location were selected.

**(B)** Graphical modeling of the predicted binding site of CTSK to the SH3 structural domain of SNX9 using alpha-fold3.

**(C-F)** Expression levels of *Col1a1* (**A**), *αsma* (**B**), *Eln* (**C**) and *F*n (**D**) in the lung tissues from rCTSK treated-mice and rCTSK treated-mice together with Pitstop2 after BLM exposure. (n=5 per group)

The data were presented as Mean ± s.d., **p*< 0.05, ***p*< 0.01, ****p* < 0.001, NS, not statistically significant.


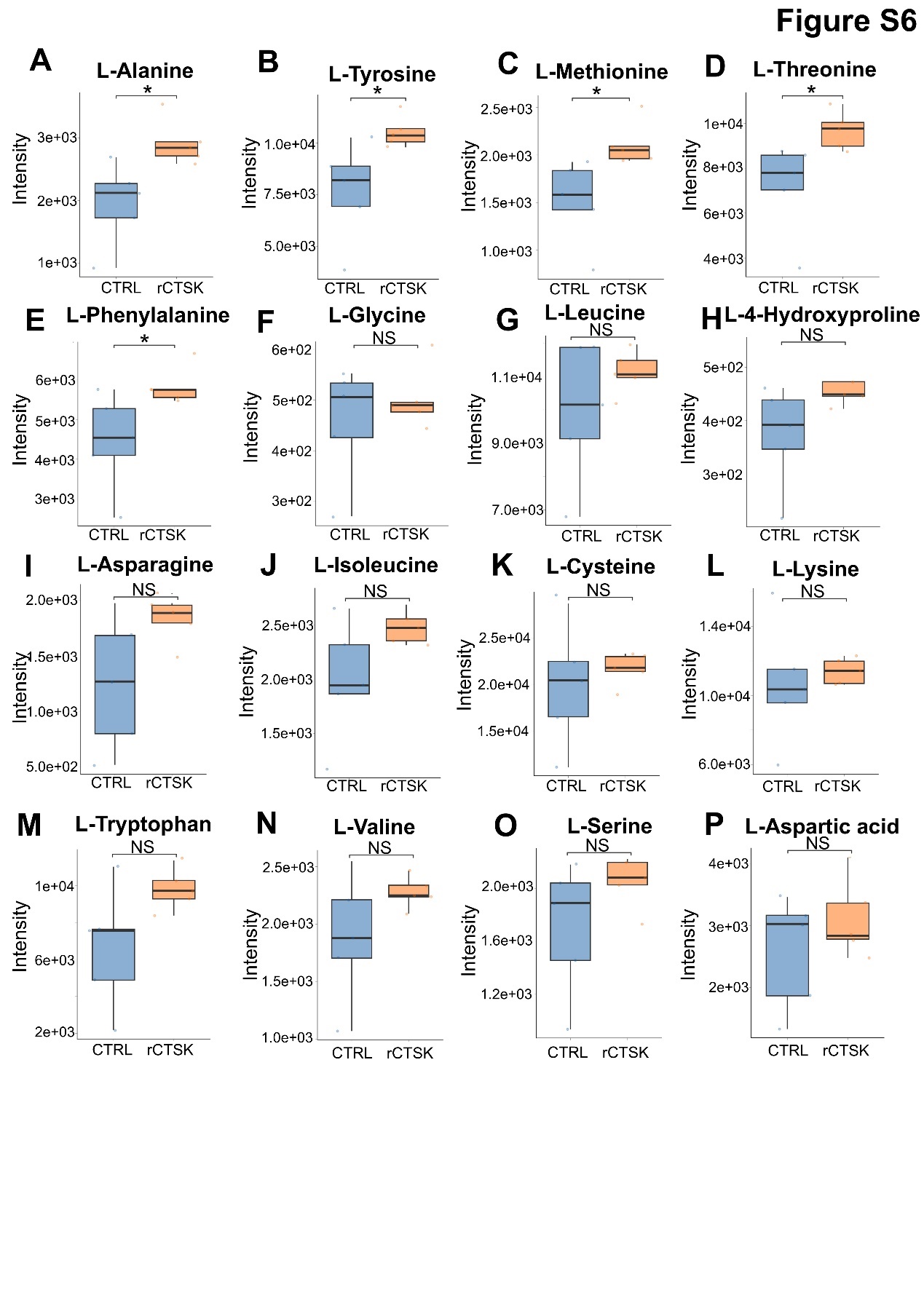


**Figure S6. Comparisons of intracellular amino acid contents in MRC-5 cells upon TGF-β1 stimulation with or without rCTSK (0.1 μg/mL) for 24 hrs by targeted Metabolomics.** (**A**) L-Alanine, (**B**) L-Tyrosine, (**C**) L-Methionine, (**D**) L-Threonine, (**E**) L-Phenylalanine, (**F**) L-Glycine, (**G**) L-Leucine, (**H**) L-4-Hydroxyproline, (**I**) L-Asparagine, (**J**) L-Isoleucine, (**K**) L-Cysteine, (**L**) L-Lysine, (**M**) L-Tryptophan, (**N**) L-Valine, (**O**) L-Serine, (**P**) L-Aspartic acid. (n=5)

The data were presented as Mean ± s.d.,**p* < 0.05. NS, not statistically significant.


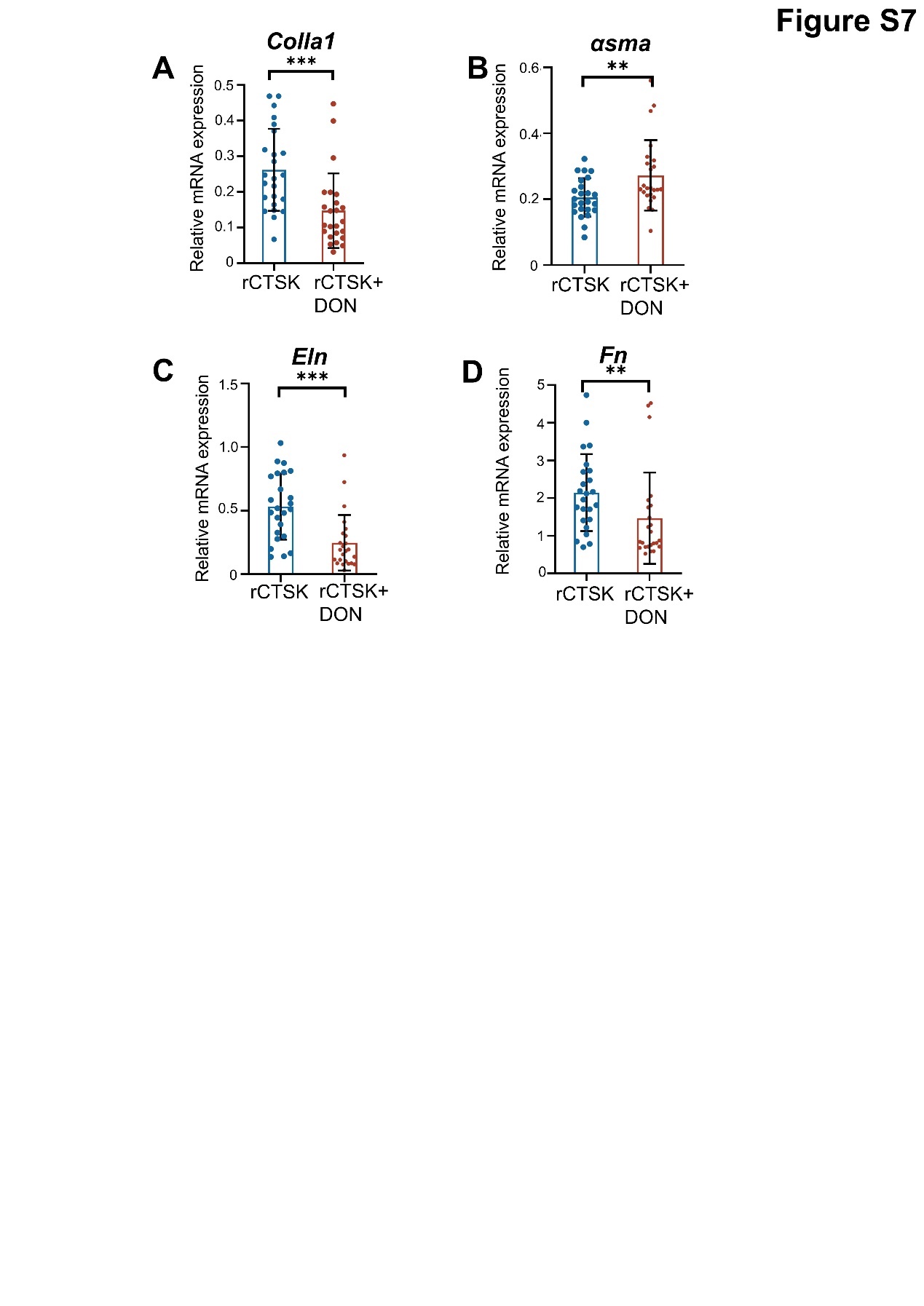


**Figure S7. (A-D)** Expression levels of *Col1a1* (**A**), *αsma* (**B**), *Eln* (**C**) and *F*n (**D**) in the lung tissues from rCTSK treated-mice and rCTSK treated-mice together with DON after BLM exposure. (n=25 per group)

The data were presented as Mean ± s.d., **p*< 0.05, ***p*< 0.01, ****p* < 0.001, NS, not statistically significant.

**Supplemental Tables 1**

| **REAGENT or RESOURCE** | **SOURCE** | **CAT** |  |
| --- | --- | --- | --- |
| **Chemicals and Recombinant Proteins** | |  |  |
| Trypsin | Gibco | 25200072 |  |
| DMEM medium | Gibco | 11965092 |  |
| DMEM medium(L-Glutamine free) | Gibco | 11960077 |  |
| FBS | Gibco | 10099141 |  |
| Penicillin streptomycin | Gibco | 15140122 |  |
| L-Glutamine (200 mM) | Gibco | 25030149 |  |
| Cathepsin K Protein, Human (His) | MedChemExpress | HY-P72156 |  |
| Cathepsin K Protein, Mouse (His) | MedChemExpress | HY-P72157 |  |
| Recombinant Human TGF-β1 | Peprotech | 100-21 |  |
| SIS3 | MedChemExpress | HY-13013 |  |
| 6-Diazo-5-oxo-L-norleucine | MedChemExpress | HY-108357 |  |
| CB839 | MedChemExpress | HY-12248 |  |
| BPTES | MedChemExpress | HY-12683 |  |
| PITSTOP2 | MedChemExpress | HY-115604 |  |
| Bleomycin | Selleck | S1214 |  |
| Odanacatib | MedChemExpress | HY-10042 |  |
| MG-132 | MedChemExpress | HY-13259 |  |
| Cycloheximide | MedChemExpress | HY-12320 |  |
| Rapamycin | MedChemExpress | HY-10219 |  |
| Odanacatib | MedChemExpress | MK-0822 |  |
| tamoxifen | Sigma-Aldrich | T5648 |  |
| collagenase type I | Sigma-Aldrich | SCR103 |  |
| **Antibody** |  |  |  |
| anti-CTSK | Santa Cruz | sc-48353 |  |
| anti-CTSK | abcam | ab187647 |  |
| anti-α-SMA | Cell Signaling Technology | 19245 |  |
| anti-COL1A1 | Cell Signaling Technology | 72026 |  |
| anti-SNX9 | Proteintech | 15721-1-AP |  |
| anti-Beta Actin | Proteintech | 66009-1-Ig |  |
| anti-SMAD3 | abcam | ab40854 |  |
| anti-SMAD3 (phospho S423 + S425) | abcam | ab52903 |  |
| anti-GLS1 | Proteintech | 12855-1-AP |  |
| anti-Beta Tubulin | Proteintech | 10094-1-AP |  |
| anti-Lamin B1 | Proteintech | 12987-1-AP |  |
| anti-Histone-H3 | Proteintech | 17168-1-AP |  |
| Anti-COL1A2 | abcam | ab308455 |  |
| Anti-COL3A1 | Proteintech | 68320-1-Ig |  |
| Anti-MYC | abmart | M20002S |  |
| Anti-FLAG | affinity | T0003 |  |
| **Oligonucleotides** |  |  |  |
| **Primers for qPCR** | **Primer（F）** | **Primer（R）** | |
| *gapd*h(Mouse),qPCR | TGACCTCAACTACATGGTCTACA | CTTCCCATTCTCGGCCTTG | |
| ctsa(Mouse),qPCR | CAGCCCTCTTTCCGGCAATA | TTTGGGTCGTTCTGCGACTC | |
| ctsb(Mouse),qPCR | CAGGCTGGACGCAACTTCTAC | TCACCGAACGCAACCCTTC | |
| ctsc(Mouse),qPCR | GTTCCCGAAGCGACATTAACT | TCGTAGGCAGTATCCAACTTCTT | |
| ctsd(Mouse),qPCR | TGGGTCCACCATAAGTACAACA | AGGCTTCCTGAGCCGTAGT | |
| ctsg(Mouse),qPCR | CCCTACATGGCATTTCTTCTGAT | GTTAGGACGAAGTCTTCTCGC | |
| ctsh(Mouse),qPCR | AAGAAAGGAAATGTTGTTTCGCC | CTTGGGCACAATCCACCAG | |
| ctsk(Mouse),qPCR | CTCGGCGTTTAATTTGGGAGA | TCGAGAGGGAGGTATTCTGAGT | |
| ctss(Mouse),qPCR | CCATTGGGATCTCTGGAAGAAAA | TCATGCCCACTTGGTAGGTAT | |
| acta2(Mouse),qPCR | CCCAGACATCAGGGAGTAATGG | TCTATCGGATACTTCAGCGTCA | |
| col1a1(Mouse),qPCR | TAAGGGTCCCCAATGGTGAGA | GGGTCCCTCGACTCCTACAT | |
| eln(Mouse),qPCR | TGTCCCACTGGGTTATCCCAT | CAGCTACTCCATAGGGCAATTTC | |
| fn(Mouse),qPCR | ATGTGGACCCCTCCTGATAGT | GCCCAGTGATTTCAGCAAAGG | |
| GAPDH(Human),qPCR | GGAGCGAGATCCCTCCAAAAT | GGCTGTTGTCATACTTCTCATGG | |
| CTSA(Human),qPCR | GTCGCCCAGAGCAATTTTGAG | TCTCCCCGGTCAGGAAAAGTT | |
| CTSB(Human),qPCR | GAGCTGGTCAACTATGTCAACA | GCTCATGTCCACGTTGTAGAAGT | |
| CTSC(Human),qPCR | CCAACTGCACCTATCTTGACC | AAGGCAAACCACTTGTAGTCATT | |
| CTSD(Human),qPCR | TGCTCAAGAACTACATGGACGC | CGAAGACGACTGTGAAGCACT | |
| CTSG(Human),qPCR | ACATGGCGTATCTTCAGATCCA | GCGCCCAGGGTGACATTTAT | |
| CTSH(Human),qPCR | CAAGTCATGGATGTCTAAGCACC | CATTGTTGTGGGCGTTTATCTTC | |
| CTSK(Human),qPCR | ACTCAAAGTACCCCTGTCTCAT | CCACAGAGCTAAAAGCCCAAC | |
| CTSS(Human),qPCR | TGACAACGGCTTTCCAGTACA | GGCAGCACGATATTTTGAGTCAT | |
| COL1A1(Human),qPCR | GAGGGCCAAGACGAAGACATC | CAGATCACGTCATCGCACAAC | |
| ACTA2(Human),qPCR | AAAAGACAGCTACGTGGGTGA | GCCATGTTCTATCGGGTACTTC | |
| GLS1(Human),qPCR | AGGGTCTGTTACCTAGCTTGG | ACGTTCGCAATCCTGTAGATTT | |
| **shRNA** | **Primer（F）** | **Primer（R）** | |
| shSNX9-1 | CCGGGCCATCCAGTAATCTCAGAAACTCGAGTTTCTGAGATTACTGGATGGCTTTTTG | AATTCAAAAAGCCATCCAGTAA  TCTCAGAAACTCGAGTTTCTGA  GATTACTGGATGGC | |
| shSNX9-2 | CCGGTCATTTCCGCATCCATTATTTCTCGAGAAATAATGGATGCGGAAATGATTTTTG | AATTCAAAAATCATTTCCGCAT  CCATTATTTCTCGAGAAATAAT  GGATGCGGAAATGA | |
